# Securitization, financial stability and effective risk retention. A European analysis

**DOI:** 10.1371/journal.pone.0228141

**Published:** 2020-02-03

**Authors:** Ana Iglesias-Casal, María-Celia López-Penabad, Carmen López-Andión, José Manuel Maside-Sanfiz

**Affiliations:** 1 Department of Quantitative Economics, Facultade de C. Económicas e Empresariais, Universidade de Santiago de Compostela, Santiago de Compostela, Spain; 2 Department of Finance and Accounting, Facultade de C. Económicas e Empresariais, Universidade de Santiago de Compostela, Santiago de Compostela, Spain; Universitat de Valencia, SPAIN

## Abstract

This paper examines the financial stability of banks that issued securitizations in the European market from 2000 to 2017. We use novel event study methodology and find that securitization has a positive impact on European banks’ systematic risk during the 2000 to 2007 period and that subsequent securitizations have not any impact on systematic risk. The increase in systematic risk is due to an increase in systemic risk and in banks’ idiosyncratic risk. By dividing the sample into those countries on the periphery and those at the core of Europe, it is found that securitization only has an impact on the systematic risk during the pre-crisis period, and only when looking at the peripheral countries does this lead to an increase in systemic risk. For individual countries, there is an observable effect for Spain and the UK prior to the crisis. On controlling for the type of collateral, it is found that this effect occurs when dealing with mortgage-based securitizations.

## 1. Introduction

Securitization is a financial technique that allows the issuer to modify a set of non-liquid rights which are subsequently traded in the market. These are therefore backed by a series of predetermined payment flows. Securitization also acts as a mechanism for transferring risk. The transactions are typically divided into different tranches with differing risk-return characteristics and a hierarchical structure; low, medium and high risk corresponding to senior, mezzanine and equity tranches respectively.

According to the European Securitization Forum, the volume of securitized assets in Europe grew from 85,998.78 million US dollars in 2000, to 1,209,250.57 million US dollars in 2008. However, during the 2007/2008 financial crisis, while European securitized assets suffered only small losses, the market itself seemed to have been tarnished by association. This provoked a sharp decline in the volume of securitizations after the first quarter of 2008. In 2013, a total of 239,599 million dollars’ worth of securitized products were issued in Europe, 80% less than in 2008. In 2014, the number of transactions slowly began to increase, reaching a volume of 288,342.63 million US dollars, before falling to 267,627.81 million US dollars in 2017. In short, the European securitization markets have remained subdued. In practice, the amount actually available to investors is even smaller, since many banks, particularly in southern Europe, are securitizing existing assets to create collateral that allows them to obtain cheap funding from the ECB. This type of securitization is most common in the UK, and then France, Italy, Spain and the Netherlands. Together these countries made up 66% of total European securitized products in 2017.

European authorities responded to the prolonged financial crisis by introducing extensive regulatory reforms. These included new rules for risk retention as well as stricter liquidity and updated capital and transparency requirements for banks intending to carry out future securitizations. The Basel Committee on Banking Supervision [1] identified a number of shortcomings in the Basel II securitization framework. Specifically, the committee underlined problems with respect to the calibration of risk weights and a lack of incentives for diligent risk management. Their analysis resulted in the specific objectives laid out in Basel III. Recently, the committee has been keen to promote the idea of simplifying securitization and making it more transparent and comparable (STC). The focus is on the long-term goal of building a sustainable securitization market rather than reviving securitization in the short term. The implementation of these regulatory initiatives has been rather fragmented and sluggish. This has given rise to uncertainty and made securitization products uneconomical for some investors [2]. The European financial system however, has received extraordinary support from the European Central Bank, which bought securitization bonds as part of its quantitative easing (QE) program.

Against this background, we analyze the impact of securitization on financial stability and the systematic and systemic risk of European securitization issuance between 2000 and 2017. We find that securitization has a positive impact on the issuing entities’ systematic risk and that the effect is present between the years 2000 and June 2007. Subsequently, no effect was recorded. The increase in systematic risk arises through an increase in systemic risk and in the specific risk for each entity. These results, for the European securitization market, were also recorded for securitization in the core and peripheral countries, and in the UK and Spain when analysed as individual countries. In the case of core countries and the UK, the increase in systematic risk was due exclusively to an increase in the specific risk of the originating entity.

There are several good reasons that justify the importance of studying the effects of securitization. First, from the point of view of the regulators, enacting relevant and soundly based regulations should help to restore market confidence in securitization. Second, this kind of study enables investors and future shareholders to better evaluate their positioning and to reduce asymmetries of information. In addition, from the point of view of the originating entities, the knowledge of how their securitizations affect their risk-taking strategy is also highly important. Finally, because of the way in which financial activity takes place, there must be a structured efficient market that offers the right levels of liquidity.

Our study enlarges upon previous empirical work in two ways. To the best of our knowledge, this is the first comprehensive study to carry out an empirical investigation into the risks arising from securitization and how these affect markets as a whole and, in particular, how they affect the originator. We carry out this analysis against the backdrop of a boom-period in securitization in Europe followed by a financial crisis and finally, a period of recovery. As [3] highlight, the data and analyses that look at European securitization is too sparse and underdeveloped to be able to draw substantive conclusions, particularly with respect to the post-crisis period.

Second, the paper enlarges upon the existing literature by using event study methodology and carrying out estimations with both symmetrical and asymmetrical event windows set around the securitization registry date. To the best of our knowledge, this is the first time asymmetrical windows have been applied to study effective risk retention. They allow us to adapt the period in which the event in question has an effect. This is a fundamental aspect of our study in which the main date, the date of the event, is that of registration; prior to this are other key dates that concern the constitution of the vehicle of securitization and the announcement of the forthcoming issue. The issue itself is made effective at later date.

This analysis makes significant advances with respect to our previous research in which we looked at the effects of securitization in Spanish financial institutions and between 1993 and 2010. The present work looks at a range of countries (Germany, Austria, Denmark, Spain, France, Netherlands, Ireland, Italy, Portugal and the UK) and analyzes the effects of securitization over a broader time period (2000–2017). This allows us to compare the effects of pre and post-crisis securitization, which is of particular importance given the dearth of studies looking into post-crisis European securitization. In addition, although the methodology used in this work is based on former studies, it has been significantly improved because it makes the estimates more flexible; as far as we know, symmetrical and asymmetrical event windows have never been used before.

Following this introduction, this paper contains five sections. Section 2 reviews the pertinent literature and the relevant empirical studies. Section 3 describes the database. The empirical methodology is set out in section 4 and the results are described in section 5. The paper concludes with a summary of the main conclusions and policy implications.

## 2. Securitization and financial stability

Importantly, securitization affects the risk profile and the financial soundness of the issuing entities and the financial system as a whole.

Economic theory provides opposing expected effects with respect to the securitization of credit risk and banking stability. This is because the expected effect has two parts, one which is direct and the other indirect. The direct impact of securitization on the risk borne by the originating entity depends on how much risk is actually transferred to external investors [4]. In this sense, the behavior of originator entities has changed over time and is determined by the type of securitization. The indirect impact of securitization on the originating entity depends on the strategy followed by the originator when reinvesting the resultant liquidity. This impact depends on the investment policy adopted and is defined by the risk transformation within the bank’s portfolio [5, 6, 7].

It is also difficult to study the effects of securitization on individual risk and upon the market as a whole because of the changes that securitization provokes in the behavior of the originators. Some of the empirical literature has raised the question whether securitization makes the further acquisition of risk more attractive for banks [8, 9, 6, 10, 11]. The appetite for risk is also related to regulatory capital arbitrage. ([3]; p. 245) state that “banks became riskier and increased systemic risk as they took advantage of securitization in order to obtain capital relief” in the years preceding the 2007–2009 crisis. Banks have often used securitization to lower costly equity capital charges in complying with the terms of regulatory requirements. Securitization enables banks to improve their capital adequacy ratios without reducing the risk of their loan portfolios. In other words, banks can securitize less risky loans and maintain the riskiest [12]. While it would seem that banks do not select and securitize corporate loans of lower credit quality, in the end, the credit quality of borrowers whose loans are securitized deteriorates by more than those in the control group [13]. It is certainly true that a securitization instrument that retains risk may induce more prudent risk behavior in banks when compared to an instrument that provides only risk transference [14, 12]. However, ([15], p. 47) affirm that “a profit maximizing bank will choose to retain the mezzanine tranche and therefore exert less screening effort. This is because due to tax incentives and mispriced government subsidies, debt is the cheaper source of capital which in turn increases the cost of equity tranche retention”.

The complexity of all these factors taken together means that there is little or no agreement as to the impact of securitization and how it affects the originators and the market as a whole, and this in spite of the vast swathe of literature that attempts to unravel the complex underlying relationships and mechanisms of the entities and mechanisms involved. The first authors to look at securitization, particularly those authors carrying out theoretical studies, ([8], [16] and [17], among others), underline a negative impact on bank lending standards and the stability of the financial system. However, prior to the crisis there was a pervasive argument for the idea that securitization stabilized the financial system since it was easier for entities to diversify, manage and allocate risk right across the economy [18, 19]. Empirical studies are also contradictory. Some indicate that the entities that carry out most securitization are also those that lend to the highest risk agents, maintain the riskiest portfolios and retain the riskiest tranches [20, 21, 22, 23]. In contrast, there are empirical studies that seem to show that securitization reduces the risk of insolvency, increases both profit and liquidity and stimulates the supply for loans ([24, 25]; among others). [18] show that these differences are in part, sometimes due to the fact that they analyze different segments of the securitization market or that they sometimes focus specifically on the USA or on European markets, with highly dissimilar structures, entities and legislation. [3] carry out a systematic, comprehensive review of the recent empirical literature on securitization, bank behaviour and financial stability and highlight that there are serious gaps in the research. Specifically, they point to the literature that looks at the effects of the post 2007–2009 crisis on the banks’ securitization behaviour, and whether and how securitization structures and pricing have changed that crisis. They state that; “it is imperative to investigate the extent to which securitization results in risk transfer during this period” (p. 251).

As mentioned above, the objective of this paper is to analyze the systematic risk of originating entities in Europe as a whole, on the periphery, within core countries, and within individual countries. The analysis aims to discover how this systematic risk might become systemic risk and undermine financial stability. There is significant cross-country variation in the European securitization markets, which stems from legal and regulatory differences. Empirical studies have thus far analyzed all countries taken together or individually, but there have not been enough observations to be able to analyze several countries individually in the same paper. We also study two groups of countries, which are differentiated according to the magnitude of the crisis they experienced, i.e. countries at core of Europe and those on the periphery. The aim of this differentiation was to discover whether the impact of securitization was different within these two groups. This is the only way to identify the differences that exist among them and to carry out an accurate assessment of the current status of the European securitization market. There are empirical studies that reference European markets which provide evidence that shows that securitization affects the risk profile of issuing entities in the pre-crisis period by increasing their systematic risk [26, 10, 9, 27, 28, 29, 30]. These studies indicate that the decrease in risk derived from securitization is counterbalanced by investments in new riskier assets. Moreover, they hypothesize that risk reduction by means of securitization is essentially determined by separating the issue into tranches. Another empirical studies, between others [31] and [32], show that credit risk securitization also has a negative impact on the issuing banks’ financial soundness.

Finally, a larger post-event beta might simply be due to the direct effect as a consequence of the fact that the first loss tranches are more likely to fail than the senior tranches. In addition, [27] study whether the increase in bank risk is due to higher individual bank risk or higher systemic risk. They find that the increase in beta is due purely to an increase in the correlation with the market (systemic risk). There may even be a decrease in the individual risk.

## 3. Data and sources

Our database is made up of 535 issues of securitization carried out in Spain, the UK, Italy, Ireland, Portugal, the Netherlands, Germany, Denmark, Austria and France between 2000 and 2017. The data was obtained from the Datastream database (Thomson Financial Services). It was supplemented with information from existing asset securitization management companies’ web pages and from the supervisory authority of the financial markets of the countries analyzed.

We utilize event study methodology, commonly used in similar types of analysis, to look at the information content of corporate events. The goal is to test for the existence of a securitization effect and to estimate its magnitude. The event study methodology employed in this paper is based on share price information and our database contains European issues carried out by listed banks. There are 63 originators who generate 535 securitization issues. We use daily closing share prices obtained from the Datastream database. Tables 1 and 2 show the volume and number of issues per year and country. As one can see, 60% of issues were carried out between 2006 and 2009, the majority of these being carried out in the UK, Spain and Italy. We highlight the UK’s higher average volume per issue.

**Table 1:** Volume and number of issues by country; Pre-crisis and during the crisis.

	Pre-crisis: 2000–2007.06		Crisis and post-crisis: 2007.07–2017	
	Volume (million €)	Number	Volume (million €)	Number
Germany	9 589.70	12	7 192.40	6
Austria	480.60	2	275.60	1
Denmark	10 513.80	4	1 088.70	2
Spain	88 029.20	89	251 975.60	126
France	2 668.10	3	6 714.90	6
Netherlands	810.10	2	18 517.86	9
Ireland			16 148.83	8
Italy	41 277.70	47	84 423.20	32
Portugal	19 278.40	16	33 234.90	25
UK	139 001.90	79	196 865.00	66
*Total*	311 649.50	254	616 437.00	281

Source: Datastream database

**Table 2:** Volume and number of issues for the whole sample for each year.

	Volume (million €)	Number
2000	2 295.90	7
2001	12 250.20	22
2002	10 327.90	18
2003	20 527.20	26
2004	29 726.70	33
2005	63 438.30	46
2006	93 278.90	67
2007	128 102.20	67
2008	163 067.90	69
2009	134 477.20	39
2010	44 270.30	21
2011	91 872.80	46
2012	36 230.80	26
2013	22 609.30	22
2014	12 545.74	8
2015	7 114.10	7
2016	55 362.30	9
2017	588.69	2
*Total*	928 086.50	535

Source: Datastream database

The study uses daily closing prices for the Eurostoxx50 Index as indicators of the market portfolio.

## 4. Empirical methodology

In this section, we describe the empirical methodology used in this study. A detailed analysis of said methodology may be found in the methodological appendix.

The first step in examining the impact of securitization on financial stability is to analyze the effect of securitization on the systematic risk of the issuing banks, an effect which we measure by using the beta coefficient. In the classic capital asset pricing model (CAPM), beta is given by the following expression:

$$\beta_{i}=\frac{Cov(R_{i},R_{m})}{\sigma_{m}^{2}}=\rho_{i,m}\frac{\sigma_{i}}{\sigma_{m}}$$ (1)

where R_i_ and R_m_ represent the returns of the banks’ assets and the market respectively; ρ_i, m_ is the Pearson correlation coefficient between the return of the stock and that of the market; σ_i_ and σ_m_ are the standard deviations of the stock’s return and that of the market respectively. Changes in beta therefore, depend on changes in the $\frac{\sigma_{i}}{\sigma_{m}}$ ratio and the correlation between the stock and market return.

In order to analyze the change in the systematic risk we adhere to the methodology set out [9] and [33]. This procedure allows the systematic risk to change while the event window is open and afterwards (in our case, the event itself corresponds to the securitization). It also makes it possible for the window to be asymmetrical around the day of the event itself, t_0_.

The resultant model that considers [T_1B_, T_2S_] asymmetric windows for which |T_1B_|>T_2S_ is as follows:

$$\begin{array}{c} R_{i,t}=\beta_{i,0}+\beta_{i,1}R_{m,t}+\\ +\beta_{i,2}[(T_{1B}-t)(t-T_{2B})D_{11,t}+(T_{1S}-t)(t-T_{2S}){C_{i}D}_{12,t}]R_{m,t}+\beta_{i,3}[(t-T_{1B})(D_{11,t}+D_{12,t})+(T_{2S}-T_{1B})D_{2,t}]R_{m,t}+\varepsilon_{i,t} \end{array}$$ (2)

where T_1B_ and T_2S_ represent the start and end of the event window; D_11,t_, D_12,t_ and D_2,t_ are the dummy variables. D_11,t_ is equal to 1 if T_1B_ ≤ t ≤ t_0_ and 0 otherwise. D_12,t_ is equal to 1 if t_0_ < t ≤ T_2S_ and 0 otherwise. D_2,t_ is equal to 1 if t > T_2S_ and 0 otherwise. *T*_2*B*_ = −*T*_1*B*_ and *T*_1*S*_ = −*T*_2*S*_ and C_i_ is a constant defined in the methodological appendix. *ε*_*i*,*t*_ is the error term and β_i_ are the coefficients that measure the systematic risk and possible changes in that risk.

In the second part, we analyze which parts of the banks’ beta correspond to their correlation with the market and which to the ratio of deviations. Subsequently, we analyze whether the possible change in systematic risk has led to a change in bank correlations. To that end, following [27], we normalize the stock and market returns using their respective standard deviations and get a series with a standard deviation of one. We implement this transformation in the following regression model, where ~ represents the transformed series:

$${\tilde{R}}_{i,t}=\rho_{i,0}+\rho_{i,1}{\tilde{R}}_{m,i,t}+\rho_{i,2}[(T_{1B}-t)(t-T_{2B})D_{11,t}+(T_{1S}-t)(t-T_{2S}){F_{i}D}_{12,t}]{\tilde{R}}_{m,i,t}+\rho_{i,3}[(t-T_{1B})(D_{11,t}+D_{12,t})+(T_{2S}-T_{1B})D_{2,t}]{\tilde{R}}_{m,i,t}+\epsilon_{i,t}$$ (3)

Where F_i_ is a constant defined in the methodological appendix and ρ_i_ are the parameters that measure the correlation between return of the stock and that of the market and possible changes or variations that may have come about because of the securitization.

Finally, the change in the ratio of standard deviations, $\frac{\sigma_{i}}{\sigma_{m}}$, is obtained as follows:

$$\Delta \frac{\sigma_{i}}{\sigma_{m}}=\frac{\sigma_{i}^{1}}{\sigma_{m}^{1}}-\frac{\sigma_{i}^{0}}{\sigma_{m}^{0}}=\frac{\beta_{i}^{0}+\Delta \beta_{i}}{\rho_{i,m}^{0}+\Delta \rho_{i,m}}-\frac{\beta_{i}^{0}}{\rho_{i,m}^{0}}$$ (4)

Where 0 indicates the period immediately prior to the event window and 1, the period immediately after.

## 5. Results

We carry out our estimations using a sample of 241 trading days symmetrically set around date of registration for the securitization with the supervisory authority of each financial market. Eq (2) is estimated by maximum likelihood while assuming that the conditional variance of error term follows a GARCH(1,1) process. I.e., equations (A6) and (A7) in the methodological appendix are jointly estimated for maximum likelihood. The returns are obtained as the logarithm of the P_t_/P_t-1_ ratio, P_t_ being the stock price. Eurostoxx50 is taken as the market portfolio index. We obtain different estimations for each securitization.

Table A1 in the S1 Appendix shows that the series of returns are stationary (augmented Dickey–Fuller [ADF] test) and follow a non-normal distribution (Jarque–Bera [JB] test). In addition, the application of the Ljung–Box test shows that the first order autocorrelation coefficients of the squared and absolute values of bank returns are significantly different from zero. This indicates the existence of volatility clustering and the expediency of jointly modeling the conditional mean and variance in order to obtain more efficient estimators.

Using event study methodology, we calculated the mean for each of the estimated coefficients in Eq (2). In order to test the significance of this measure we use both a parametric and a non-parametric test. The former is the t-test which is valid when there is normality. The latter is the Wilcoxon signed rank [Wilcoxon SR] test, which is applied to the median and is more suitable in the absence of normality which is the case here. The results are completed with the number and percentage of the coefficients, which are significantly different from zero at the 10% significance level.

### 5.1. Results for the entire sample

We used different lengths of both symmetrical and asymmetrical windows to carry out the analysis. [33] state that the most important feature of the estimated event window is its temporal location since small windows tend to miss important economic effects, while larger windows can bias results by combining abnormal returns from the event period with those that are external to it. [9] use symmetrical 21-day windows and, in order to control for robustness 11 and 41 day event periods. We use both symmetrical and asymmetrical windows. The former have durations of 31, 21 and 11 days. The asymmetrical windows use different combinations that begin at 15 or 10 days prior to the event. These windows always had longer periods of time prior to the event than after it. The number of days that the windows include after the date of the event varies between 14 and 5. It is therefore logical that, prior to the date we consider—the date of the event, i.e. the official registration of the issue, the market had heard about the securitization issuance. This would have occurred through the constitution of a special purpose vehicle or announcement for the issue for example. This would have affected the risk of the originating entity for longer than the post-registration period.We also used 3 different time periods, 2000 to 2017, 2000 to June 2007 and July 2007 to 2017. A summary of the most relevant results is given in Table 3. To control for the robustness of our baseline regression we also carried out the analysis for other windows of different length -the results are available to those interested on request-. The first part of these findings shows the results for all the European securitizations which correspond to the symmetrical window [-15,+15] and the whole period. The results for the analyzed windows are similar and thus we only comment the results for the [-15,+15] and [-15, +5] windows as these are fairly representative.

**Table 3:** Summary results: Europe.

**Period 2000–2017**		n = 535					
**window -+15**				Wilcoxon SR test		coeff sig 10%	
	mean	t-test	p-value		p-value	number	percentage
β_0_	-0.01399	-2.473	0.014	2.567	0.010	99	18.5
β_1_	0.80509	36.883	0.000	19.765	0.000	466	87.1
β_2_	0.00008	0.770	0.441	0.122	0.903	125	23.4
β_3_	0.00150	3.527	0.001	4.167	0.000	201	37.6
α_0_	0.70727	9.981	0.000	19.941	0.000	357	66.7
α_1_	0.19935	14.893	0.000	18.216	0.000	429	80.2
α_2_	0.60332	40.138	0.000	19.245	0.000	426	79.6
**β**	0.80509						
**Δβ**	0.04512						
**window -15+5**				Wilcoxon SR test		coeff sig 10%	
	mean	t-test	p-value		p-value	number	percentage
β_0_	-0.01361	-2.394	0.017	2.635	0.008	98	18.3
β_1_	0.80541	36.962	0.000	19.760	0.000	471	88.0
β_2_	0.00008	0.672	0.502	0.274	0.784	108	20.2
β_3_	0.00187	3.092	0.002	3.525	0.000	211	39.4
α_0_	0.70643	10.868	0.000	19.931	0.000	358	66.9
α_1_	0.19635	15.480	0.000	18.179	0.000	435	81.3
α_2_	0.59864	39.257	0.000	19.242	0.000	419	78.3
**β**	0.80541						
**Δβ**	0.03736						
**Pre-crisis**	n = 254						
**window -+15**				Wilcoxon SR test		coeff sig 10%	
	mean	t-test	p-value		p-value	number	percentage
β_0_	0.01065	1.716	0.087	1.640	0.101	47	18.5
β_1_	0.55969	22.646	0.000	13.401	0.000	202	79.5
β_2_	-0.00018	-1.373	0.171	1.933	0.053	48	18.9
β_3_	0.00235	4.320	0.000	4.437	0.000	86	33.9
α_0_	0.56413	4.391	0.000	13.742	0.000	179	70.5
α_1_	0.22215	10.104	0.000	12.303	0.000	202	79.5
α_2_	0.55268	23.576	0.000	12.826	0.000	194	76.4
**β**	0.55969						
**Δβ**	0.07062						
**window -15+5**				Wilcoxon SR test		coeff sig 10%	
	mean	t-test	p-value		p-value	number	percentage
β_0_	0.01160	1.902	0.058	1.813	0.070	39	15.4
β_1_	0.56231	22.953	0.000	13.465	0.000	203	79.9
β_2_	0.00004	0.276	0.783	0.412	0.680	46	18.1
β_3_	0.00313	3.978	0.000	4.191	0.000	87	34.3
α_0_	0.57412	4.517	0.000	13.746	0.000	176	69.3
α_1_	0.21527	10.926	0.000	12.653	0.000	195	76.8
α_2_	0.55385	24.930	0.000	12.999	0.000	189	74.4
**β**	0.56231						
**Δβ**	0.06258						

For the -/+15 window, all European securitizations and the period 2000 to 2017, the coefficients that measure the change in systematic risk are significantly different from zero in 125 cases for β_2_ and in 201 cases for β_3_, representing 23.4% and 37.6% of all the estimated coefficients respectively. However, the average for the β_2_ is not significantly different from zero, in contrast to the average for β_3_ which is.

In the conditional variance equation, 80% of α_1_ values and 79.6% of α_2_ are significant, which confirms the use of a GARCH(1,1). A similar situation is obtained in all of the estimations carried out as is shown in Tables from 3 to 10 of the results.

**Fig 1:**
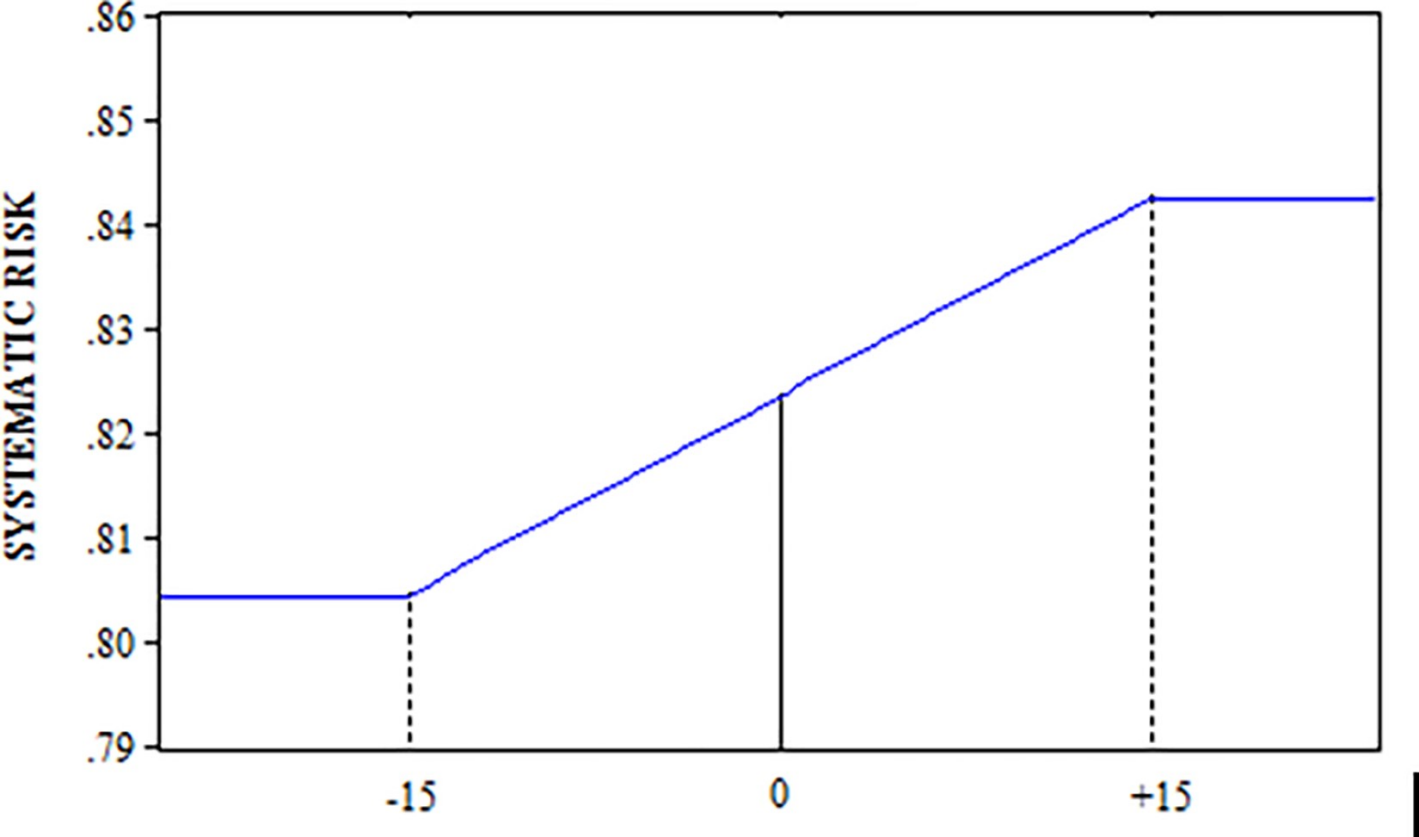
Systematic risk ([-15,+15] window). Fig 1 Displays the evolution of the systematic risk within the window [-15, +15] centered on the registry date. This information is based on the results of Table 3, obtained using 535 securitizations.

The mean of the estimated β_1_ coefficients before the event window is 0.8051. The mean of the β_2_ parameters is not significant but the β_3_ (0.0015) is. The β_3_ value means that the systematic risk grows during the event window while the value for β_2_ indicates linear growth. At the end of the window, systematic risk is equal to 0.8502 (Fig 1). This implies that the average increase in systematic risk is 0.0451 during the event window.

We assume that the response of returns to securitization issues, during and after the event period, completely reflects the economic effect of securitizations on the originating bank’s systematic risk. The results do not differ substantially among the different windows. Therefore, even if the daily change in systematic risk during the window varies as a function of its size, the post-event beta resulting from the accumulated change is highly similar in all cases: 0.8428 and 0.8502 for the[-15,+5] and [-15,+15] windows respectively.

**Fig 2:**
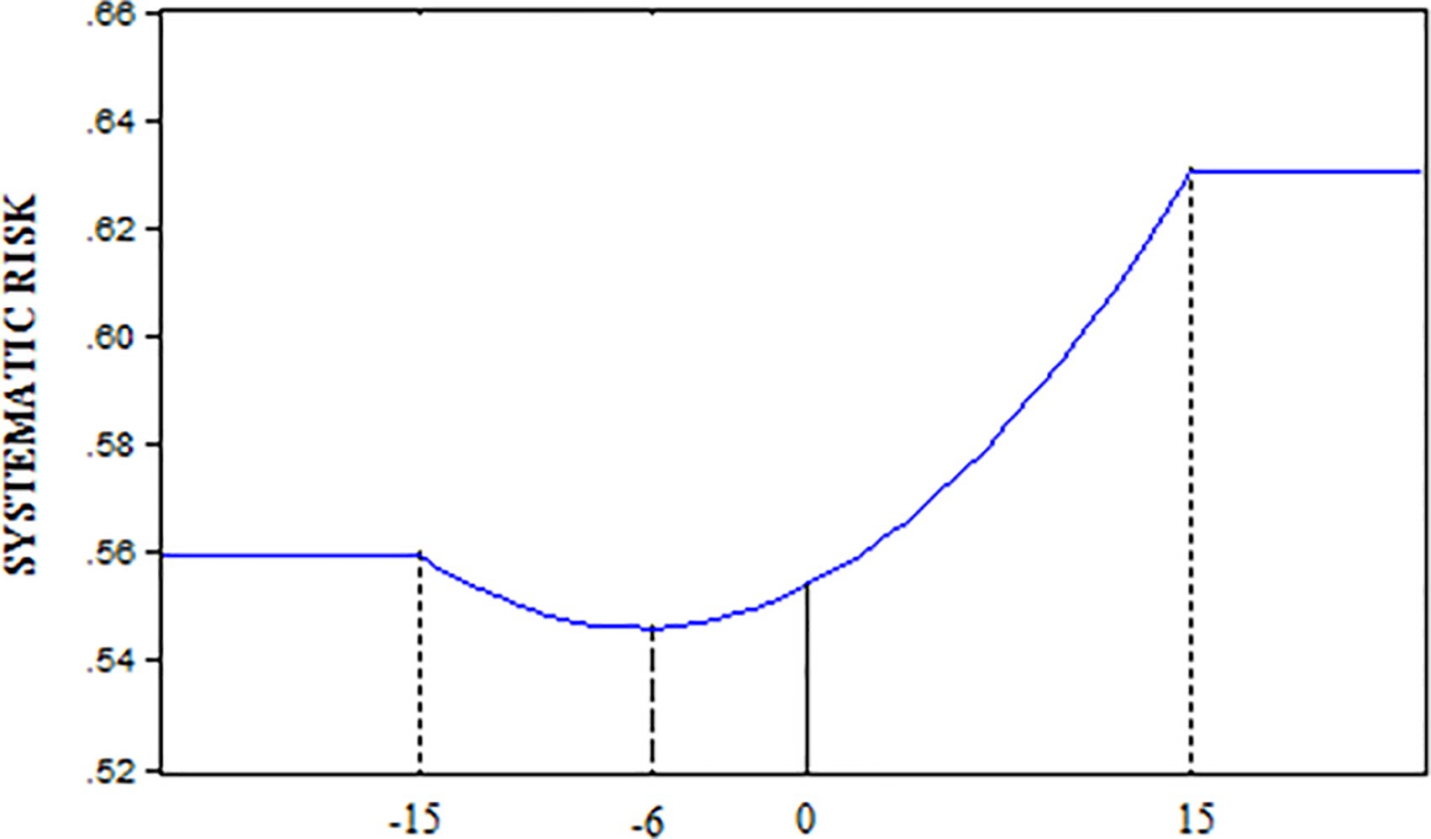
Systematic risk pre-crisis ([-15,+15] window). Fig 2 Displays the evolution of the systematic risk within the window [-15, +15] centered on the pre-crisis registry date. This information is based on the results of Table 3, obtained using 254 securitizations.

We then repeat the analysis by dividing the sample into two periods, from 2000 to June 2007 and from July 2007 to 2017. A summary of the results is given in Table 3 (above) for the [-15,+15] window and the pre-crisis period. As one can see, until June 2007, the parameters β_2_ and β_3_ are significantly different from zero by 18.9% and 33.9% respectively, for all of the coefficients estimated. The changes in systematic risk were sufficiently great to produce a significant mean in the case of β_2_ and β_3_. The mean of the estimated β_1_ coefficients before the event window is 0.5597. The mean of β_2_ is negative (-0.00018) and significant, which means that the evolution of systematic risk during the event window follows a quadratic function, a convex run of systematic risk, and reaches its minimum six days prior to the registration date (Fig 2); from that moment on its value increases. The systematic risk is 0,6303 at the end of the event window given that there is a significant value of 0.0024 for β_3_. Therefore, the main change in systematic risk within the window is 0.0706.

For the crisis and post crisis periods, changes in systematic risk measured within β_2_ and β_3_ are insufficient to produce a mean that differs significantly from zero. Therefore, the change in systematic risk is not significant. The definitive implementation of Basel II from 2007 implies that the volume of all the retained tranches would determine the regulatory minimum capital requirement. This dynamic is in line with our results, as we do not register a significant change in the systematic risk from 2007.

The average of the β_1_ coefficients for the whole period, i.e. the average beta pre-event for the entire period, is 0.8051, for the pre-crisis period 0.5597 and for the crisis period 1.0424. This would clearly seem to indicate that the systematic risk of entities is higher during the crisis than prior to it. It may also be true that securitization contributed to this increase in systematic risk during the pre-crisis period. The results for the crisis period are not included in the paper because the change in systematic risk is not significant. However, they are available on request.

As discussed in section 4, we also performed the analysis for asymmetrical windows and results do not differ substantially from those obtained for symmetrical windows. Only in the period 2000 to June 2007 with asymmetrical windows is the β_2_ coefficient non-significant, implying a linear trend in the rise in the value of beta. The linear trend in the increase in beta is also the behaviour observed in the analyses by groups and by single countries when the increase in systematic risk is significant. The results for other windows and periods are similar. Table 3 shows a summary of the results for the entire period and from 2000 to June 2007 and the [-15,+5] window.

The results of our estimations show an increase in the systematic risk of the European banks during the event window. Initially, given that securitization is a vehicle that enables risk transference, this might look unusual. Nevertheless, when the tail risk of the senior tranches (the least risky) is lower than the risk of default in the first losses tranches retained by the originator, there is little or zero probability of risk transmission. In addition, the post event systematic risk increases when the liquidity of the originator is reinvested in riskier assets (indirect effect). This scenario is perfectly possible in European markets since banks were permissive in providing credit and the requisites and regulations in place prior to the crisis were less demanding. Even the reinvestment of liquidity in a diversified portfolio might have led to an increase in beta when the pre-event value is lower than 1, as occurs in our analysis. If the diversification of the bank’s portfolio increases, its performance becomes more closely allied to that of the market and, by extension, its beta will tend to 1. In addition, the systematic risk might increase if the liquidity is used to modify the structure of the capital. This could be in the form of paying higher dividends, or repurchasing their own stocks, which might lead to an increase in leverage and a consequent increase in risk.

Finally, Eqs (3) and (4) allow us to determine the part of the increase in beta that is due to the correlation effect and that which is due to the quotient for the standard deviations of the banks in relation to the market. For the pre-crisis period and the [-15,+15] window, Table 4 shows that the average increase in ρ is 0.022. In addition, the positive result obtained for Δ(σi/σm) equal to 0.0926 indicates that one part of the rise in beta is due to a convergence in the correlations between the originators and the market and the other is due to an increase in bank-specific risk. This means that the growth in beta that takes place as the outcome of securitization gives rise to an increase in bank correlations and specific risk for each entity. We find the same result for asymmetric windows. For the whole period, the increase in ρ is non-significant; therefore, we cannot conclude that risk was transferred to the market. Thus, the increase in beta is due only to an increase in bank-specific risk.

**Table 4:** Summary results–normalized: Europe.

**Period 2000–2017**		n = 535					
**window -+15**				Wilcoxon SR test		coeff sig 10%	
	mean	t-test	p-value		p-value	number	percentage
ρ_0_	-0.00560	-2.112	0.035	2.073	0.038	88	16.5
ρ_1_	0.53513	44.682	0.000	19.843	0.000	465	86.9
ρ_2_	-0.00007	-1.420	0.156	0.898	0.369	83	15.5
ρ_3_	0.00006	0.263	0.792	0.723	0.470	158	29.5
α_0_	0.20808	21.830	0.000	20.036	0.000	331	61.9
α_1_	0.17114	14.495	0.000	17.342	0.000	389	72.7
α_2_	0.55084	34.131	0.000	18.623	0.000	383	71.6
**ρ**	0.53513						
**Δρ**	0						
**window-15+5**						coeff sig 10%	
	mean	t-test	p-value	Wilcoxon SR test	p-value	number	percentage
ρ_0_	-0.00687	-2.626	0.009	2.549	0.011	88	16.5
ρ_1_	0.53606	44.906	0.000	19.870	0.000	470	87.9
ρ_2_	0.00001	0.245	0.807	1.136	0.256	89	16.6
ρ_3_	0.00001	-0.030	0.976	0.075	0.940	156	29.2
α_0_	0.20246	21.513	0.000	20.038	0.000	333	62.2
α_1_	0.17268	15.315	0.000	17.613	0.000	389	72.7
α_2_	0.54984	34.092	0.000	18.674	0.000	388	72.5
**ρ**	0.53606						
**Δρ**	0						
**Pre-crisis**	n = 254						
**window+-15**						coeff sig 10%	
	mean	t-test	p-value	Wilcoxon SR test	p-value	number	percentage
ρ_0_	0.0075	1.894	0.059	1.580	0.114	45	17.8
ρ_1_	0.4342	24.987	0.000	13.575	0.000	201	79.5
ρ_2_	-0.0002	-2.558	0.011	2.187	0.029	38	15.0
ρ_3_	0.0007	2.069	0.040	2.357	0.018	77	30.4
α_0_	0.2614	14.986	0.000	13.813	0.000	167	66.0
α_1_	0.1788	9.478	0.000	11.443	0.000	183	72.3
α_2_	0.5116	19.948	0.000	12.294	0.000	182	71.9
**ρ**	0.4342						
**Δρ**	0.0220						
**window-15+5**						coeff sig 10%	
	mean	t-test	p-value	Wilcoxon SR test	p-value	number	percentage
ρ_0_	0.00787	2.062	0.040	1.855	0.064	40	15.8
ρ_1_	0.43233	24.632	0.000	13.552	0.000	204	80.3
ρ_2_	-0.00002	-0.234	0.815	0.414	0.679	44	17.3
ρ_3_	0.00116	2.265	0.024	2.537	0.011	85	33.5
α_0_	0.25595	14.570	0.000	13.814	0.000	166	65.4
α_1_	0.17812	9.251	0.000	11.677	0.000	186	73.2
α_2_	0.51161	19.950	0.000	12.294	0.000	185	72.8
**ρ**	0.43233						
**Δρ**	0.02316						

In light of the findings, we can affirm that securitization gave rise to a growth in global or systemic risk within the European financial system in the pre-crisis period but not during and after the crisis period.

We have included a control analysis of the type of underlying asset, which differentiates between mortgages, business loans and others. For the period prior to the crisis, in the issues that contain mortgage and business loan collateral, an increase in systematic risk is registered. This increase is not significant for securitizations with other types of collateral. On carrying out the decomposition, we find that, when the underlying collateral consists of business loans, the increase in ρ is not significant at the 5% level. This contrasts with mortgage securitization where the increase in ρ is significant, and ranges between 0.0293 and 0.0183 depending on the window. We can affirm that the increase in the pre-crisis systematic risk led to an increase in the correlation with the market and the specific risk of the originators in the case of mortgage securitizations. In the case of lending to companies however, this only resulted in an increase in the specific risk. The results are available upon request from the authors.

### 5.2. Results of core vs. periphery

The following stage involves carrying out the analysis for two groups, which we labelled core and periphery. The former group contained Germany, Austria, Denmark, France, Netherlands and UK; and the latter, Portugal, Ireland, Italy and Spain. This second group is made up of countries for whom the effects of the crisis were deeper and lasted longer than the rest of Europe. These countries are located on the periphery and characterized by the fragility of their economies.

Table 5 provides an overview of the results for peripheral countries. Irrespective of the window used, on average, securitization during the period immediately prior to the crisis produces an increase in systematic risk. This behaviour is also observed when analysing the whole period, although the increase in risk is less pronounced. However, in the period prior to the crisis, this phenomenon is not present.

**Table 5:** Summary results: Periphery.

**Period 2000–2017** n = 343							
**window -+15**				Wilcoxon SR test		coefs sig 10%	
	mean	t-test	p-val		p-val	number	percentage
β_0_	-0.03444	-4.692	0.000	4.764	0.000	72	21.0
β_1_	0.76068	28.175	0.000	15.675	0.000	291	84.8
β_2_	0.00011	0.925	0.356	0.115	0.908	84	24.5
β_3_	0.00120	2.289	0.023	2.608	0.009	131	38.2
α_0_	0.63194	7.561	0.000	15.990	0.000	225	65.6
α_1_	0.20571	11.877	0.000	15.093	0.000	282	82.2
α_2_	0.61399	34.859	0.000	15.635	0.000	272	79.3
β	0.76068						
Δβ	0.03606						
**window -15+5**				Wilcoxon SR test		coefs sig 10%	
	mean	t-test	p-val		p-val	number	percentage
β_0_	-0.03467	-4.752	0.000	4.818	0.000	71	20.7
β_1_	0.76414	28.246	0.000	15.687	0.000	293	85.4
β_2_	0.00005	0.342	0.732	0.654	0.513	76	22.2
β_3_	0.00126	1.692	0.092	1.883	0.060	142	41.4
α_0_	0.68032	7.866	0.000	15.979	0.000	222	64.7
α_1_	0.20285	12.259	0.000	15.139	0.000	282	82.2
α_2_	0.60821	33.846	0.000	15.564	0.000	272	79.3
**β**	0.76414						
**Δβ**	0.02522						
**Pre-crisis**	n = 152						
**window -+15**				Wilcoxon SR test		coefs sig 10%	
	mean	t-test	p-val		p-val	number	percentage
β_0_	-0.00295	-0.321	0.749	0.141	0.888	28	18.4
β_1_	0.50638	15.718	0.000	10.210	0.000	118	77.6
β_2_	-0.00016	-0.912	0.363	1.783	0.075	29	19.1
β_3_	0.00275	3.551	0.001	3.411	0.001	63	41.5
α_0_	0.42989	4.985	0.000	10.692	0.000	106	69.7
α_1_	0.25378	7.412	0.000	10.131	0.000	123	80.9
α_2_	0.55163	19.674	0.000	10.212	0.000	114	75.0
**β**	0.50638						
**Δβ**	0.08238						
**window -15+5**				Wilcoxon SR test		coefs sig 10%	
	mean	t-test	p-val		p-val	número	percentage
β_0_	-0.00174	-0.194	0.846	0.128	0.898	27	17.8
β_1_	0.50781	15.899	0.000	10.258	0.000	121	79.6
β_2_	0.00001	-0.040	0.968	0.924	0.355	30	19.7
β_3_	0.00357	3.163	0.002	3.233	0.001	67	44.1
α_0_	0.45265	5.072	0.000	10.692	0.000	106	69.7
α_1_	0.25083	7.737	0.000	10.228	0.000	122	80.3
α_2_	0.54896	19.312	0.000	10.157	0.000	114	75.0
**β**	0.50781						
**Δβ**	0.07144						

On analysing whether or not the increase in systematic risk may result in an increase in the correlation with market behaviour (Table 6), it may be observed that there is only a significant increase in ρ for the period prior to the crisis. The average increase for the +/- 15 window is equal to 0.0287. A Δ(*σ*_*i*_/*σ*_*m*_) score of 0.106 shows that one part of the increase goes towards an increase in bank correlations and the other to an increase in the specific risk for each entity. However, for the [-15,+5] window, there is only a Δρ which is significant at the 10% level.

**Table 6:** Summary results-normalized: Periphery.

**Period 2000–2017**		n = 343					
**window -+15**				Wilcoxon SR test		coefs sig 10%	
	mean	t-test	p-val		p-val	number	percentage
ρ_0_	-0.01315	-3.807	0.000	3.646	0.000	64	18.7
ρ_1_	0.52572	33.466	0.000	15.811	0.000	292	85.1
ρ_2_	-0.00005	-0.869	0.385	0.723	0.469	57	16.6
ρ_3_	0.00016	0.558	0.577	0.698	0.485	96	28.0
α_0_	0.20053	17.072	0.000	16.045	0.000	210	61.2
α_1_	0.18222	12.166	0.000	14.710	0.000	256	74.6
α_2_	0.54701	28.291	0.000	15.064	0.000	242	70.6
**ρ**	0.52572						
**Δρ**	0						
**window-15+5**						coefs sig 10%	
	mean	t-test	p-val	Wilcoxon SR test	p-val	number	percentage
ρ_0_	-0.01419	-4.133	0.000	3.976	0.000	67	19.5
ρ_1_	0.52810	33.555	0.000	15.851	0.000	297	86.6
ρ_2_	-0.00006	-0.831	0.407	0.070	0.944	64	18.7
ρ_3_	0.00007	0.184	0.854	0.019	0.985	95	27.7
α_0_	0.19460	16.838	0.000	16.048	0.000	219	63.85
α_1_	0.17983	12.694	0.000	14.797	0.000	261	76.09
α_2_	0.54928	28.161	0.000	15.067	0.000	249	72.6
**ρ**	0.52810						
**Δρ**	0						
**Pre-crisis**	n = 152						
**window+-15**						coefs sig 10%	
	mean	t-test	p-val	Wilcoxon SR test	p-val	number	percentage
ρ_0_	0.0042	0.775	0.439	0.630	0.529	27	17.8
ρ_1_	0.4094	18.119	0.000	10.434	0.000	118	77.6
ρ_2_	-0.0002	-2.069	0.040	2.004	0.045	22	14.5
ρ_3_	0.0010	2.093	0.038	2.319	0.020	42	27.6
α_0_	0.2282	13.287	0.000	10.694	0.000	93	61.2
α_1_	0.2100	7.274	0.000	9.844	0.000	112	73.7
α_2_	0.5415	19.384	0.000	10.169	0.000	112	73.7
**ρ**	0.40944						
**Δρ**	0.0287						
**window-15+5**						coefs sig 10%	
	mean	t-test	p-val	Wilcoxon SR test	p-val	number	percentage
ρ_0_	0.00358	0.660	0.510	0.591	0.554	26	17.1
ρ_1_	0.41434	18.205	0.000	10.477	0.000	124	81.6
ρ_2_	-0.00014	-1.231	0.220	1.294	0.196	22	14.5
ρ_3_	0.00085	1.345	0.181	1.658	0.097	37	24.3
α_0_	0.23082	12.870	0.000	10.694	0.000	94	61.8
α_1_	0.20998	7.357	0.000	9.842	0.000	111	73.0
α_2_	0.52487	17.562	0.000	9.939	0.000	111	73.0
**ρ**	0.41434						
**Δρ**	0.01698						

Tables 7 and 8 provide an overview of the results for core countries. As with the peripheral countries, the core countries exhibit an increase in systematic risk for the period immediately prior to the crisis, but not during the subsequent period. The main difference resides in the fact that there is no transmission of risk to the market. This can be seen in Table 8, in which the normalized results show that ρ_3_ is not significant. Therefore, it may only be asserted that the increase in systematic risk has led to an increase in idiosyncratic bank risk. There may be many reasons that explain this difference. The fact that their economies were stronger, the financial crisis less virulent in these countries, and the support received by financial institutions, are almost certainly three of the most important factors that prevented the transmission of increased risk to the market place.

**Table 7:** Summary results: Core.

**Period 2000–2017**		n = 192					
**window -+15**				Wilcoxon SR test		coefs sig 10%	
	mean	t-test	p-val		p-val	number	percentage
β_0_	0.02255	2.777	0.006	2.385	0.017	27	14.1
β_1_	0.88443	24.260	0.000	11.995	0.000	175	91.2
β_2_	0.00002	0.132	0.895	0.268	0.789	41	21.4
β_3_	0.00204	2.801	0.006	3.465	0.001	70	36.5
α_0_	0.84185	6.528	0.000	11.941	0.000	132	68.8
α_1_	0.18798	9.012	0.000	9.990	0.000	147	76.6
α_2_	0.58426	21.127	0.000	11.237	0.000	154	80.2
**β**	0.88443						
**Δβ**	0.06132						
**window -15+5**				Wilcoxon SR test		coefs sig 10%	
	mean	t-test	p-val		p-val	number	percentage
β_0_	0.02401	2.870	0.005	2.294	0.022	27	14.1
β_1_	0.87914	24.257	0.000	11.979	0.000	178	92.7
β_2_	0.00012	0.711	0.478	0.396	0.692	32	16.7
β_3_	0.00295	2.872	0.005	3.347	0.001	69	35.9
α_0_	0.75308	7.953	0.000	11.931	0.000	136	70.8
α_1_	0.18475	9.523	0.000	9.805	0.000	153	79.7
α_2_	0.58155	20.873	0.000	11.295	0.000	147	76.6
**β**	0.87914						
**Δβ**	0.05904						
**Pre-crisis**	n = 102						
**window -+15**				Wilcoxon SR test		coefs sig 10%	
	mean	t-test	p-val		p-val	number	percentage
β_0_	0.01596	1.998	0.048	1.566	0.117	14	13.7
β_1_	0.62782	17.080	0.000	8.733	0.000	86	84.3
β_2_	-0.00008	-0.420	0.675	0.023	0.981	22	21.6
β_3_	0.00214	2.981	0.004	3.251	0.001	26	25.5
α_0_	0.43471	8.175	0.000	8.676	0.000	73	71.6
α_1_	0.18776	8.167	0.000	7.267	0.000	80	78.4
α_2_	0.54504	15.017	0.000	8.159	0.000	72	70.6
**β**	0.62782						
**Δβ**	0.06429						
**window -15+5**				Wilcoxon SR test		coefs sig 10%	
	mean	t-test	p-val		p-val	número	percentage
β_0_	0.01723	2.127	0.036	1.819	0.069	16	15.7
β_1_	0.62359	16.706	0.000	8.696	0.000	89	87.3
β_2_	0.00016	0.811	0.419	0.461	0.645	11	10.8
β_3_	0.00346	3.568	0.001	3.552	0.000	23	22.6
α_0_	0.44865	8.192	0.000	8.676	0.000	73	71.6
α_1_	0.18859	8.123	0.000	7.144	0.000	80	78.4
α_2_	0.54934	16.177	0.000	8.426	0.000	72	70.6
**β**	0.62359						
**Δβ**	0.06916						

**Table 8:** Summary results–normalized: Core.

**Period 2000–2017**		n = 192					
**window -+15**				Wilcoxon SR test		coefs sig 10%	
	mean	t-test	p-val		p-val	number	percentage
ρ_0_	0.00791	2.043	0.042	1.632	0.103	24	12.5
ρ_1_	0.55194	30.584	0.000	11.976	0.000	173	90.1
ρ_2_	-0.00005	-1.219	0.224	0.574	0.566	26	13.5
ρ_3_	-0.00011	-0.270	0.788	0.277	0.782	62	32.3
α_0_	0.22156	13.611	0.000	12.015	0.000	121	63.0
α_1_	0.15133	7.919	0.000	8.868	0.000	133	69.3
α_2_	0.55768	19.324	0.000	10.966	0.000	141	73.4
**ρ**	0.55194						
**Δρ**	0						
**window-15+5**						coefs sig 10%	
	mean	t-test	p-val	Wilcoxon SR test	p-val	number	percentage
ρ_0_	0.00620	1.643	0.102	1.283	0.199	21	10.9
ρ_1_	0.55027	30.948	0.000	11.970	0.000	173	90.1
ρ_2_	0.00015	1.676	0.095	1.955	0.051	25	13.0
ρ_3_	-0.00016	-0.275	0.784	0.030	0.976	61	31.8
α_0_	0.21651	13.399	0.000	12.015	0.000	114	59.38
α_1_	0.15992	8.583	0.000	9.174	0.000	128	66.67
α_2_	0.55084	19.361	0.000	11.047	0.000	139	72.4
**ρ**	0.55027						
**Δρ**	0						
**Pre-crisis**	n = 102						
**window+-15**						coefs sig 10%	
	mean	t-test	p-val	Wilcoxon SR test	p-val	number	percentage
ρ_0_	0.0092	1.543	0.126	1.198	0.231	15	14.7
ρ_1_	0.4677	17.800	0.000	8.683	0.000	84	82.4
ρ_2_	-0.0001	-1.138	0.258	0.808	0.419	12	11.8
ρ_3_	0.0005	0.913	0.363	1.025	0.305	30	29.4
α_0_	0.2793	11.453	0.000	8.766	0.000	66	64.7
α_1_	0.1591	7.004	0.000	6.673	0.000	69	67.7
α_2_	0.4984	12.645	0.000	7.865	0.000	68	66.7
**ρ**	0.46766						
**Δρ**	0.0000						
**window-15+5**						coefs sig 10%	
	mean	t-test	p-val	Wilcoxon SR test	p-val	number	percentage
ρ_0_	0.00826	1.428	0.156	1.108	0.268	13	12.8
ρ_1_	0.46278	17.788	0.000	8.663	0.000	84	82.4
ρ_2_	0.00015	1.238	0.219	0.951	0.341	12	11.8
ρ_3_	0.00074	1.034	0.304	1.165	0.244	29	28.4
α_0_	0.26414	10.918	0.000	8.766	0.000	63	61.8
α_1_	0.15606	6.973	0.000	6.803	0.000	67	65.7
α_2_	0.52250	13.254	0.000	7.965	0.000	70	68.6
**ρ**	0.46278						
**Δρ**	0						

### 5.3. Results by country

We now undertake an analysis of three individual countries; the UK, Spain and Italy. These are countries with large volumes of securitization, which means there is enough data to carry out a specific analysis. There are 145 securitizations for the UK, 215 for Spain and 79 for Italy, out of a total of 535. The remaining countries (France, the Netherlands, Portugal, Austria, Denmark, Germany and Ireland) are dealt with together because the number of existing securitizations for each would substantially weaken the results.

#### 5.3.1. The United Kingdom

Table 9 provides an overview of the results for the UK. For the [-15,+15] window and for the whole period, the mean of the estimated β_1_ coefficients before the event window (systematic risk) is 0.822. The systematic risk is 0.8924 at the end of the event window because of a significant β_3_ value of 0,0021. The results for the [-15,+5] window are similar to those described above.

**Table 9:** Summary results: UK.

**Period 2000–2017**		n = 145					
**window+-15**				Wilcoxon SR test		coeff sig 10%	
	mean	t-test	p-value		p-value	number	percentage
β_0_	0.02396	2.532	0.012	1.944	0.052	19	13.1
β_1_	0.82166	19.949	0.000	10.418	0.000	130	89.7
β_2_	0.00007	0.280	0.780	0.024	0.981	34	23.5
β_3_	0.00212	2.612	0.010	3.166	0.002	49	33.8
α_0_	0.94335	5.660	0.000	10.441	0.000	97	66.9
α_1_	0.19605	7.758	0.000	8.872	0.000	114	78.6
α_2_	0.56206	16.822	0.000	9.545	0.000	117	80.7
**β**	0.82894						
**Δβ**	0.06345						
**window-15+5**				Wilcoxon SR test		coeff sig 10%	
	mean	t-test	p-value		p-value	number	percentage
β_0_	0.02580	2.611	0.010	1.786	0.074	19	13.1
β_1_	0.81386	20.004	0.000	10.400	0.000	132	91.0
β_2_	0.00022	1.042	0.299	0.823	0.411	24	16.6
β_3_	0.00301	2.691	0.008	3.146	0.002	44	30.3
α_0_	0.84185	6.980	0.000	10.445	0.000	101	69.7
α_1_	0.19465	8.380	0.000	8.736	0.000	117	80.7
α_2_	0.55455	16.719	0.000	9.620	0.000	109	75.2
**β**	0.83416						
**Δβ**	0.06012						
**Pre-crisis**	n = 79						
**window+-15**				Wilcoxon SR test		coeff sig 10%	
	mean	t-test	p-value		p-value	number	percentage
β_0_	0.02353	2.683	0.009	2.167	0.030	13	16.5
β_1_	0.57261	14.346	0.000	7.548	0.000	63	79.8
β_2_	-0.00030	-1.258	0.212	0.755	0.450	17	21.5
β_3_	0.00239	3.919	0.000	3.365	0.001	16	20.3
α_0_	0.47401	6.425	0.000	7.709	0.000	56	70.9
α_1_	0.18554	8.543	0.000	6.517	0.000	64	81.0
α_2_	0.52856	12.374	0.000	7.128	0.000	59	74.7
**β**	0.57261						
**Δβ**	0.07158						
**window-15+5**				Wilcoxon SR test		coeff sig 10%	
	mean	t-test	p-value		p-value	number	percentage
β_0_	0.02545	2.946	0.004	2.578	0.010	10	12.7
β_1_	0.57434	14.691	0.000	7.573	0.000	63	79.8
β_2_	0.00007	0.242	0.809	0.130	0.897	9	11.4
β_3_	0.00329	4.173	0.000	3.814	0.000	14	17.7
α_0_	0.51008	6.899	0.000	7.719	0.000	55	69.6
α_1_	0.18602	8.737	0.000	6.747	0.000	62	78.5
α_2_	0.48298	10.517	0.000	6.668	0.000	55	69.6
**β**	0.57434						
**Δβ**	0.06572						

For the pre-crisis period and for both symmetrical and asymmetrical windows, the results again coincide with those mentioned above. However, β_1_ now has a lower value (0.57), which increases by approximately 0.07. The resultant score for the final systematic risk is 0.64, which is below the final figure reached for the entire period. During the crisis and afterwards the changes in systematic risk are non-significant for all of the windows analyzed.

Below, Table 10 shows a summary of the normalized results. The coefficient ρ_3_ is not significant for any of the periods analyzed and we cannot therefore draw any conclusions concerning the transfer of risk to the market, except that the increase in systematic risk is due to an increase in idiosyncratic bank risk.

**Table 10:** Summary of results–normalized: UK.

**Period 2000–2017**		n = 145					
**window -+15**				Wilcoxon SR test		coeff sig 10%	
	mean	t-test	p-value		p-value	number	percentage
ρ_0_	0.00740	1.655	0.100	1.123	0.261	17	11.7
ρ_1_	0.51931	25.302	0.000	10.392	0.000	128	88.3
ρ_2_	-0.00015	-1.463	0.146	1.099	0.272	21	14.5
ρ_3_	-0.00013	-0.276	0.783	0.241	0.810	50	34.5
α_0_	0.23806	11.948	0.000	10.445	0.000	90	62.1
α_1_	0.15828	6.993	0.000	7.913	0.000	102	70.3
α_2_	0.53809	15.097	0.000	9.208	0.000	108	74.5
**ρ**	0.51931						
**Δρ**	0						
**window-15+5**						coeff sig 10%	
	mean	t-test	p-value	Wilcoxon SR test	p-value	number	percentage
ρ_0_	0.00690	1.559	0.121	1.113	0.266	15	10.3
ρ_1_	0.51864	25.695	0.000	10.394	0.000	128	88.3
ρ_2_	0.00012	1.162	0.247	1.289	0.197	14	9.7
ρ_3_	-0.00029	-0.431	0.667	0.140	0.889	50	34.5
α_0_	0.23323	11.897	0.000	10.445	0.000	81	55.9
α_1_	0.16400	7.550	0.000	8.181	0.000	96	66.2
α_2_	0.53666	15.592	0.000	9.385	0.000	106	73.1
**ρ**	0.51864						
**Δρ**	0						
**Pre-crisis**	n = 79						
**window+-15**						coeff sig 10%	
	mean	t-test	p-value	Wilcoxon SR test	p-value	number	percentage
ρ_0_	0.01211	1.848	0.068	1.444	0.149	12	15.2
ρ_1_	0.43764	14.990	0.000	7.582	0.000	63	79.8
ρ_2_	-0.00023	-1.698	0.093	1.400	0.162	10	12.7
ρ_3_	0.00056	0.943	0.349	1.156	0.248	24	30.4
α_0_	0.28600	9.779	0.000	7.719	0.000	51	64.6
α_1_	0.14750	7.122	0.000	5.750	0.000	55	69.6
α_2_	0.49899	10.798	0.000	6.835	0.000	53	67.1
**ρ**	0.43764						
**Δρ**	0						
**window-15+5**						coeff sig 10%	
	mean	t-test	p-value	Wilcoxon SR test	p-value	number	percentage
ρ_0_	0.01152	1.838	0.070	1.576	0.115	11	8.9
ρ_1_	0.43529	14.568	0.000	7.558	0.000	62	78.5
ρ_2_	0.00003	0.212	0.833	0.100	0.920	8	10.1
ρ_3_	0.00085	0.983	0.329	1.205	0.228	26	32.9
α_0_	0.28242	9.812	0.000	7.714	0.000	51	64.6
α_1_	0.14819	7.107	0.000	5.896	0.000	57	72.2
α_2_	0.49519	10.458	0.000	6.805	0.000	54	68.4
**ρ**	0.43529						
**Δρ**	0						

Besides not belonging to the Eurozone, the United Kingdom may be considered to be a special case, since its securitization market has been run by UK entities at the forefront of financial innovation and might therefore considered to be a world research centre for securitization. The close-knit relationship with another of the big global financial hubs (New York) gave rise to the immediate spread of the repercussions of the crisis already affecting the British originators. The interrelatedness of these two financial centers is what eventually gave rise to the UK’s financial problems and not the low quality of the credit in the UK. It is curious that in our work, despite recording an increase in the systematic risk of the originating banks, there is no significant correlation with the market and therefore this increase only corresponds to an increase in the specific risk of the originator.

#### 5.3.2. Spain

Table 11 shows the summarized results for Spain. For the entire period, both for the [-15,+15] and the [-15,+ 5] windows, there is an observable increase in systematic risk as a consequence of securitization, given that β_3_ is significant. The means of the estimated β_1_ coefficients before the event windows are 0.7928 and 0.7956 respectively. Immediately after the window, systematic risk has a value of 0.829 and 0.8214 for the [-15,+15] and [-15,+ 5] windows respectively.

**Table 11:** Summary results: Spain.

**Period 2000–2017**		n = 215					
**window+-15**				Wilcoxon SR test		coeff sig 10%	
	mean	t-test	p-value		p-value	number	percentage
β_0_	-0.03095	-4.204	0.000	3.693	0.000	48	22.3
β_1_	0.79281	23.234	0.000	12.337	0.000	181	84.2
β_2_	0.00008	0.632	0.528	0.026	0.980	52	24.2
β_3_	0.00121	2.310	0.022	2.492	0.013	82	38.1
α_0_	0.40622	8.194	0.000	12.644	0.000	140	65.1
α_1_	0.15821	13.709	0.000	11.808	0.000	175	81.4
α_2_	0.61976	26.607	0.000	12.305	0.000	167	77.7
**β**	0.79281						
**Δβ**	0.03615						
**window-15+5**				Wilcoxon SR test		coeff sig 10%	
	mean	t-test	p-value		p-value	number	percentage
β_0_	-0.03186	-4.324	0.000	3.753	0.000	45	20.9
β_1_	0.79559	23.364	0.000	12.351	0.000	180	83.7
β_2_	0.00004	0.230	0.818	0.358	0.721	45	20.9
β_3_	0.00129	1.674	0.096	1.928	0.054	92	42.8
α_0_	0.44400	8.152	0.000	12.628	0.000	138	64.2
α_1_	0.15790	13.560	0.000	11.879	0.000	177	82.3
α_2_	0.61614	26.258	0.000	12.260	0.000	169	78.6
**β**	0.79559						
**Δβ**	0.02576						
**Pre-crisis**	n = 89						
**window+-15**				Wilcoxon SR test		coeff sig 10%	
	mean	t-test	p-value		p-value	number	percentage
β_0_	0.00968	1.161	0.249	1.137	0.255	16	18.0
β_1_	0.56369	12.344	0.000	7.720	0.000	69	77.5
β_2_	-0.00014	-0.845	0.401	1.231	0.218	15	16.9
β_3_	0.00258	3.204	0.002	2.778	0.006	36	40.5
α_0_	0.30100	6.835	0.000	8.191	0.000	67	75.3
α_1_	0.19367	8.776	0.000	7.593	0.000	76	85.4
α_2_	0.52460	12.162	0.000	7.238	0.000	66	74.2
**β**	0.56369						
**Δβ**	0.07740						
**window-15+5**				Wilcoxon SR test		coeff sig 10%	
	mean	t-test	p-value		p-value	number	percentage
β_0_	0.01000	1.226	0.223	1.281	0.200	16	18.0
β_1_	0.56391	12.466	0.000	7.798	0.000	69	77.5
β_2_	0.00009	0.361	0.719	0.135	0.893	17	19.1
β_3_	0.00329	2.816	0.006	2.443	0.015	37	41.6
α_0_	0.25779	8.315	0.000	8.191	0.000	64	71.9
α_1_	0.18956	8.906	0.000	7.794	0.000	71	79.8
α_2_	0.58552	17.057	0.000	7.941	0.000	68	76.4
**β**	0.56391						
**Δβ**	0.06578						

On dividing the sample into two periods, the pre-crisis period and the crisis and post-crisis period, the above results hold for the former, and there are no longer significant changes in systematic risk during the latter. One can also see that in the pre-crisis period, the mean of the β_1_ coefficients is lower than the mean for the whole period. Increases were recorded that reached 0.6411 and 0.6297 for the [-15,+15] and [-15,+5] windows, scores that were lower than those registered for the whole period.

The increase in systematic risk in Spain was probably due to the “originate to hold” type of securitization that was prevalent in the country. There is, therefore, no real risk transfer since the final loss for the portfolio is lower than that for the first-loss tranche. From the perspective of the indirect effect, the growth in systematic risk might have been produced by the reinvestment of the liquidity in assets of lower credit quality, exacerbated by an expansion in the amount of credit available. These results coincide with those obtained [30].

Table 12 provides the normalized results for the whole period with a symmetric window and the pre-crisis period with symmetric and asymmetric windows. In the three scenarios the ρ_3_ coefficient is significant and reflects increases in ρ of 0.02, 0.04 and 0.03 respectively. The results obtained for the Δ(σ_i_/σ_m_) are 0.011, 0.0556 and 0.0594 respectively, indicating that the increase in beta is due to an increase in the correlation between the originators and the market and an increase in the specific risk of banks, particularly in the pre-crisis period.

**Table 12:** Summary results–normalized: Spain.

**Period 2000–2017**		n = 215					
**window+-15**						coeff sig 10%	
	mean	t-test	p-value	Wilcoxon SR test	p-value	number	percentage
ρ_0_	-0.007969	-2.875	0.004	2.550	0.011	41	19.1
ρ_1_	0.564081	26.654	0.000	12.436	0.000	179	83.3
ρ_2_	-1.12E-05	-0.153	0.879	0.381	0.704	34	15.8
ρ_3_	0.000705	2.288	0.023	2.158	0.031	54	25.1
α_0_	0.192655	12.596	0.000	12.710	0.000	136	63.3
α_1_	0.139079	15.241	0.000	11.534	0.000	160	74.4
α_2_	0.543131	21.143	0.000	11.745	0.000	148	68.8
**ρ**	0.564081						
**Δρ**	0.021150						
**window-15+5**						coeff sig 10%	
	mean	t-test	p-value	Wilcoxon SR test	p-value	number	percentage
ρ_0_	-0.014165	-3.306	0.001	2.989	0.003	42	19.5
ρ_1_	0.572265	27.444	0.000	12.516	0.000	184	85.6
ρ_2_	-0.0000545	-0.620	0.536	0.208	0.835	41	19.1
ρ_3_	0.000443	1.0398	0.299	0.433	0.665	52	24.2
α_0_	0.186840	12.627	0.000	12.708	0.000	138	64.2
α_1_	0.139839	15.102	0.000	11.636	0.000	162	75.4
α_2_	0.545163	20.878	0.000	11.736	0.000	154	71.6
**ρ**	0.572265						
**Δρ**	0						
**Pre-crisis**	n = 89						
**window+-15**						coeff sig 10%	
	mean	t-test	p-value	Wilcoxon SR test	p-value	number	percentage
ρ_0_	0.007743	1.099	0.275	1.313	0.189	16	18.2
ρ_1_	0.447051	13.620	0.000	7.954	0.000	66	75.0
ρ_2_	-1.38E-04	-1.164	0.248	0.888	0.375	14	15.9
ρ_3_	0.001332	2.522	0.014	2.516	0.012	24	27.3
α_0_	0.2456	9.208	0.000	8.191	0.000	64	72.73
α_1_	0.150322	9.364	0.000	7.332	0.000	69	78.41
α_2_	0.491998	10.970	0.000	7.013	0.000	66	75
**ρ**	0.447051						
**Δρ**	0.039900						
**window-15+5**						coeff sig 10%	
	mean	t-test	p-value	Wilcoxon SR test	p-value	number	percentage
ρ_0_	0.00864	1.315	0.192	1.448	0.148	13	14.6
ρ_1_	0.45096	13.763	0.000	7.990	0.000	69	77.5
ρ_2_	-0.00008	-0.515	0.608	0.736	0.462	19	21.4
ρ_3_	0.00149	1.960	0.053	2.070	0.038	27	30.3
α_0_	0.23177	8.892	0.000	8.191	0.000	60	67.4
α_1_	0.14263	10.084	0.000	7.487	0.000	69	77.5
α_2_	0.53514	12.827	0.000	7.434	0.000	69	77.5
**ρ**	0.45096						
**Δρ**	0.02978						

The decomposition of the beta coefficient shows that there has been an increase in the idiosyncratic risk, but that risk has also been transmitted to the market, increasing systemic risk (ρ).

#### 5.3.3. Italy

The results summarized for Italy are shown in Table 13. As we can see, in contrast to the UK and Spain there is no increase in systematic risk at the 5% significance level. For symmetrical windows, the systematic risk increases over the period of the event window only at a 10% significance level, and this follows a linear function. In this case, the pre- and post-event betas in the pre-crisis period show levels of 0.4859 and 0.5864, which are lower than the betas for the total period (0.7157) and (0.7869).

**Table 13:** Summary results: Italy.

**Period 2000–2014**		n = 79					
**window+-15**				Wilcoxon SR test		coeff sig 10%	
	mean	t-test	p-value		p-value	number	percentage
β_0_	-0.04747	-2.540	0.013	2.739	0.006	16	20.3
β_1_	0.71567	13.490	0.000	7.636	0.000	67	84.8
β_2_	0.00020	0.613	0.542	0.560	0.576	15	19.0
β_3_	0.00238	1.578	0.119	1.723	0.085	31	39.2
α_0_	1.39103	3.262	0.002	7.690	0.000	50	63.3
α_1_	0.30147	4.921	0.000	7.157	0.000	60	76.0
α_2_	0.59895	16.121	0.000	7.475	0.000	62	78.5
**β**	0.71567						
**Δβ**	0.07125						
**window-15+5**				Wilcoxon SR test		coeff sig 10%	
	mean	t-test	p-value		p-value	number	percentage
β_0_	-0.04222	-2.341	0.022	2.739	0.006	14	17.7
β_1_	0.72949	13.707	0.000	7.651	0.000	68	86.1
β_2_	0.00039	0.985	0.328	0.736	0.462	22	27.9
β_3_	0.00248	1.108	0.272	1.307	0.191	39	49.4
α_0_	1.37915	3.263	0.002	7.690	0.000	49	62.0
α_1_	0.28543	5.353	0.000	7.411	0.000	60	76.0
α_2_	0.59847	16.551	0.000	7.499	0.000	62	78.5
**β**	0.72949						
**Δβ**	0						
**Pre-crisis**	n = 47						
**window+-15**				Wilcoxon SR test		coeff sig 10%	
	mean	t-test	p-value		p-value	number	percentage
β_0_	-0.01076	-0.479	0.634	1.159	0.247	10	21.3
β_1_	0.48591	9.658	0.000	5.783	0.000	36	76.6
β_2_	0.00001	0.026	0.980	0.143	0.886	7	14.9
β_3_	0.00335	1.734	0.090	1.847	0.065	19	40.4
α_0_	1.40942	2.119	0.040	5.952	0.000	27	57.5
α_1_	0.32678	3.502	0.001	5.635	0.000	34	72.3
α_2_	0.58557	11.669	0.000	5.730	0.000	35	74.5
**β**	0.48591						
**Δβ**	0.10053						
**window-15+5**				Wilcoxon SR test		coeff sig 10%	
	mean	t-test	p-value		p-value	number	percentage
β_0_	-0.00735	-0.332	0.742	1.169	0.242	9	19.2
β_1_	0.49743	9.915	0.000	5.815	0.000	36	76.6
β_2_	0.00011	0.213	0.833	0.238	0.812	10	21.3
β_3_	0.00408	1.415	0.164	1.540	0.124	21	44.7
α_0_	1.43187	2.176	0.035	5.952	0.000	26	55.3
α_1_	0.29725	3.739	0.001	5.794	0.000	34	72.3
α_2_	0.57805	11.889	0.000	5.751	0.000	35	74.5
**β**	0.49743						
**Δβ**	0						

Table 14 shows normalized results only in those cases in which the increase in systematic risk is significant at the 10% level. The ρ_3_ coefficient is non-significant in both of the cases in which there was increased systematic risk, either for the whole period or for the pre-crisis period. Therefore, we cannot draw any conclusions as to the transfer risk to the market.

**Table 14:** Summary results–normalized: Italy.

**Period 2000–2014**		n = 79					
**window+-15**				Wilcoxon		coeff sig 10%	
	mean	t-test	p-value	signed rank	p-value	number	percentage
ρ_0_	-0.016435	-2.309	0.024	2.793	0.005	14	18.0
ρ_1_	0.468822	16.853	0.000	7.665	0.000	67	85.9
ρ_2_	1.89E-06	0.013	0.990	0.159	0.874	15	19.2
ρ_3_	-0.000227	-0.276	0.783	0.130	0.897	29	37.2
α_0_	0.218203	7.143	0.000	7.695	0.000	45	57.69
α_1_	0.250749	4.853	0.000	6.810	0.000	57	73.08
α_2_	0.568853	14.279	0.000	7.318	0.000	58	74.36
**ρ**	0.468822						
**Δρ**	0						
**Pre-crisis**	n = 47						
**window+-15**				Wilcoxon		coeff sig 10%	
	mean	t-test	p-value	signed rank	p-value	number	percentage
ρ_0_	-0.007064	-0.701	0.487	1.296	0.195	10	21.3
ρ_1_	0.36943	10.478	0.000	5.847	0.000	36	76.6
ρ_2_	-4.67E-06	-0.022	0.983	-0.005	0.996	10	21.3
ρ_3_	0.001101	0.997	0.324	1.307	0.191	17	36.2
α_0_	0.277433	6.011	0.000	5.952	0.000	22	46.81
α_1_	0.277634	3.467	0.001	5.275	0.000	31	65.96
α_2_	0.517643	9.175	0.000	5.445	0.000	32	68.09
**ρ**	0.36943						
**Δρ**	0						

Italian banks show a lower propensity for financial innovation. Securitization in Italy has never been a widespread financial operation as in other countries, such as the US, the UK and Spain [34]. Indeed, in the main, Italian banks have used customer deposits to finance their loan positions while the Italian securitization market itself has been concentrated in just a few Italian banks. The nature of Italian securitization has changed to a certain extent too. The amount of defaults as a proportion of total securitizations decreased over time after 2005. In Italy, the supervisory authority has taken a very cautious approach to securitization: banks may securitize primarily to facilitate turnover in the loan portfolio and to increase funding, and much less as a vehicle of risk transfer. As a consequence, the impact of the financial crisis on the Italian banking system was very limited. Finally, the fact that Italian banks were less active on international markets meant that they were less exposed to the worst hit financial markets. According to [35] the subprime mortgage market is only a small segment of the credit market (representing close to zero in the EU and less than 10% of all securitized mortgages in the US). In Italy, the subprime part of the market has remained relatively undeveloped because of an inherently cautious approach to securitization. In Italy, banks tend to securitize loans with specific characteristics, particularly those that are less opaque [23]. [35] reveal that during the 1995–2006 period, around two thirds of all equity tranches were directly retained by the originating banks (11% for the mezzanine and 4% for the senior tranches, respectively). Italian banks tend to securitize relatively good quality loans, choosing how much risk to keep depending on the characteristics of the transaction; they keep a higher proportion of the risk for themselves when the loans are of better quality. This is consistent with our results in which there is no increase in systematic risk and no risk transfer to the market.

#### 5.3.4. Other countries

There are fewer available estimates for the remaining countries; France, the Netherlands, Portugal, Austria, Denmark, Germany and Ireland. Hence, we deal with these countries as a single group. There were 96 securitizations out of a total of 535 on the database for this group. The peculiarities of each country with regard to the securitization market means that treating them as a whole conceals the effects that securitization may have on each of these countries.

The results of the analysis for the different types and lengths of windows and for the different periods were not generally significant. Therefore, we found no significant increase in systematic risk in the event window, making an analysis of risk transfer to the market unnecessary.

## 6. Conclusions

We analyzed a sample of 535 securitizations issued by 63 European financial entities from 2000 to 2017. The event analysis methodology allowed us to examine how systematic risk changed gradually within symmetrical and asymmetrical event windows. We find that securitization has caused an increase in the issuing entities’ systematic risk within the 2000 to June 2007 period and that there is no significant change in systematic risk thereafter.

Similarly, the systematic risk of the originators before the event is greater during the crisis than in the pre-crisis or whole period. In the pre-crisis period, the initial systematic risk is at its lowest levels and gradually increases in a linear fashion.

The split between core and peripheral countries indicates that, in both groups, the systematic risk prior to the crisis increases because of securitizations, but no significant changes are observed from 2007 onwards. The above results are also recorded for securitizations issued in the UK and Spain, and for Italy too, but only for symmetrical windows.

The increase in systematic risk is concentrated in the pre-crisis period and arises through an increase in bank correlations (systemic risk) and in the specific risk for each entity. This effect, which was recorded for all of the European securitization issues and for the peripheral countries, was also present in the Spanish market but not in the core group and in the UK, where the increase in beta was due exclusively to an increase in the specific risk of the originating entity. On controlling for the type of collateral, we can affirm that the increase in systematic and systemic risk only takes place in the case of mortgage securitizations.

Undoubtedly, the cross-country, legal and regulatory differences in securitization markets, and even in the financial systems themselves, strongly influenced the economic behavior of individual countries and their banking entities in different ways prior to the financial crisis. The way Italian originators dealt with risk, limiting market securitization to high-quality loans and an “originate-to-hold” model, is perfectly captured by the methodology we use in this analysis. In contrast, the Spanish market was oriented towards retaining riskier tranches on the balance sheet together with a tendency to reinvest the cash flows from securitization in products that did not diversify the originator’s portfolio. This explains the increase in systematic risk caused both by a closer correlation with the market and a greater exposure to risk for each individual entity. The UK market, which is similar to the US Anglo-Saxon model, clearly experienced an increase in systematic risk, but we cannot conclude that this risk was transferred to the market.

In general, until 2007, all primary issues were placed with final investors and other banks; post 2007, almost all deals were retained by the originating banks and many were used as collateral with central banks. As a result, and given that this procedure was common to all countries, the transmission of risk was no longer possible.

Our results are consistent with those obtained by other authors such as [26], [10], [9], [27] and [30], who show that the transfer of credit risk has important effects on bank risk. Our results add value in the sense that we not only analyze the period before the crisis, but the entire crisis period and the subsequent recovery. We find that there was no increase in systemic risk during the crisis periods and recovery, in contrast to the period directly preceding the crisis. The lengthy timeframe used in our study has allowed us to control for changes in the dynamic of securitization over time and to be able to state that the securitization structures have changed since the crisis. Further, and in contrast to other studies, we conclude that, in the pre-crisis period, a bank’s increase in systematic risk is due to higher individual bank risk and higher systemic risk, and that these effects come about when the securitizations are based on mortgage collateral. Furthermore, we also conduct individual analyses for several countries, enabling us to carry out a comparison, hitherto impossible in similar studies. We have also developed a novel methodology that allows us to apply asymmetrical windows in order to detect relevant fluctuations financial stocks prior to registration. This methodology could be utilized to analyze a whole range of corporate events.

Our result is highly significant for all of those collectives that have links to securitization, i.e. the originators, investors, future shareholders, policy makers, rulers and pundits. Securitization has great potential for providing any market with liquidity and, as a consequence, improving the functioning of these markets. However, due to the very nature of these markets, the information that each of the participants has access to is different. This gives rise to conflicts and frictions, the consequences of which are sometimes grave. We consider that there are three aspects addressed in our work that represent an advance in the path towards greater transparency of the securitization market in Europe: the change in securitization structures after the financial crisis and the consequent change in the associated risk; the analysis of these changes according to geographic location; and the prevalence of risk transmission to the market in mortgage structures. It is hoped that this study will contribute to greater transparency in the European securitization market while mitigating the relative paucity of empirical literature on Europe after the crisis.

## Supporting information

S1 Appendix (DOCX)

S1 Data (XLSX)
