# Supplementary material for: Securitization, financial stability and effective risk retention. A European analysis
Source: PLoS One. 2020 Feb 3;15(2):e0228141. doi: 10.1371/journal.pone.0228141 (PMC6996833; doi:10.1371/journal.pone.0228141)
Supplement: S1 Appendix — (DOCX) [file pone.0228141.s001.docx]

Appendix

Methodological appendix

Equation (1) allows us to break down the systematic risk (beta factor) into two; the correlation between the stock and market returns (ρ_i, m_) and the relative standard deviation of the return of the banks’ stocks (σ_i_/σ_m_). Therefore, changes in beta may be due to changes in each one of the components.

In order to implement the first phase of the work i.e. the calculation of the systematic risk and the extent to which it might vary, we take as our starting point equations (1), (2), (3) and (5) set out in the work of Uhde and Michalak (2010). We introduce variants that allow us to adjust the analysis so that we can use asymmetric windows around the event day, t_0_. The window is denoted by [T_1B_, T_2S_] for which |T_1B_|>T_2S._

The lack of availability of information prior to the registry date has led us to propose a more general methodology as a variant of the previous model that considers [T_1B_, T_2S_] asymmetric windows for which |T_1B_|>T_2S_. The model is as follows:

$R_{i,t}=\beta_{i,0}+\beta_{i,1}R_{m,t}+\Delta^{1st part event}\beta_{i,1}D_{11,t}R_{m,t}+\Delta^{2nd part event}\beta_{i,1}D_{12,t}R_{m,t}+\Delta^{after}\beta_{i,1}D_{2,t}R_{m,t}+\eta_{i,t}$ (A1)

where R_i_ and R_m_ are the returns of the banks’ stocks and the market respectively and D_11,t_, D_12,t_ and D_2,t_ are the dummy variables. D_11,t_ is equal to 1 if T_1B_ ≤ t ≤ t_0_ and 0 otherwise. D_12,t_ is equal to 1 if t_0_ < t ≤ T_2S_ and 0 otherwise. D_2,t_ is equal to 1 if t > T_2S_ and 0 otherwise. t_0_ The equations used are:

$\Delta^{1st part event}\beta_{i,1}=\beta_{i,2}\left( T_{1B}-t \right)\left( t-T_{2B} \right)+\beta_{i,3}\left( t-T_{1B} \right)+\nu_{i,t}$ (A2)

$\Delta^{2nd part event}\beta_{i,1}=\beta_{i,2}\left( T_{1S}-t \right)\left( t-T_{2S} \right)C_{i}+\beta_{i,3}\left( t-T_{1B} \right)+\mu_{i,t}$ (A3)

$\Delta^{after}\beta_{i,1}=\beta_{i,3}\left( T_{2S}-T_{1B} \right)+\xi_{i,t}$ (A4)

where $T_{2B}=-T_{1B}$ and $T_{1S}=-T_{2S}$ and

$C_{i}=\frac{\frac{\beta_{i,3}^{2}}{4\beta_{i,2}}+\beta_{i,2}T_{2B}^{2}+\beta_{i,3}T_{2B}}{\frac{\beta_{i,3}^{2}}{4\beta_{i,2}}+\beta_{i,2}T_{2S}^{2}+\beta_{i,3}T_{2B}}$ (A5)

such that
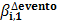
$\Delta^{1st part event}\beta_{i,1}$ and $\Delta^{2nd part event}\beta_{i,1}$ measure the marginal change in systematic risk between the start of the window and the day of the event, and between the day of the event and the end of the window respectively. If β_i_,_2_ is negative, the change in the systematic risk is a convex function during the window, if it is positive, the function will be concave. When β_i_,_2_ is zero, the function will be linear. The C_i_ term is introduced so that the quadratic functions that are part of Equations (A2) and (A3) have a common maximum (minimum). If the window is symmetric, C_i_ =1.

Equation (A4) describes $\Delta^{after}\beta_{i,1}$
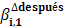
 as the marginal change in systematic risk after the event window.

Substituting the above equations into Equation (A1) leads to the following regression model:

$R_{i,t}= \beta_{i,0}+\beta_{i,1}R_{m,t}+$

$+\beta_{i,2}\left[ \left( T_{1B}-t \right)\left( t-T_{2B} \right)D_{11,t}+\left( T_{1S}-t \right)\left( t-T_{2S} \right){C_{i}D}_{12,t} \right]R_{m,t}+\beta_{i,3}\left[ \left( t-T_{1B} \right)(D_{11,t}+D_{12,t})+\left( T_{2S}-T_{1B} \right)D_{2,t} \right]R_{m,t}+\varepsilon_{i,t}$ (A6)

In the above equation, the error term ε_i,t_ is a function of market return, with a)
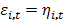
$\varepsilon_{i,t}=\eta_{i,t}$ over the period of the pre-event estimation, b) $\varepsilon_{i,t}=\eta_{i,t}+\upsilon_{i,t}R_{m,t}$
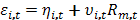
 during the first part of the window, c) $\varepsilon_{i,t}=\eta_{i,t}+\mu_{i,t}R_{m,t}$
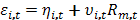
 during the second part of the window and d) $\varepsilon_{i,t}=\eta_{i,t}+\xi_{i,t}R_{m,t}$ within
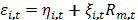
 the post-event period. In this work, we assumed that the error term ε_i,t_ is characterised by conditional heteroscedasticity and is modelled using a GARCH(1,1):

$h_{i,t}=\alpha_{i,0}+\alpha_{i,1}{\varepsilon_{i,t}}^{2}+\alpha_{i,2}h_{i,t-1}$
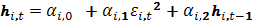
 (A7)

where $h_{i,t}=E({\varepsilon_{i,t}}^{2}/\Omega_{t-1})$
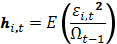
 is the conditional variance of ε_i,t_.

Furthermore, following Equation (1), we identify that part of the bank’s beta that corresponds to its correlation with the market and that which corresponds to the relative standard deviation. Subsequently, we estimate whether the possible change in systematic risk has led to a change in bank correlations.

In order to carry out this, the second part of our analysis of the impact of securitization on financial stability, we normalize the stocks and market returns by dividing these by their respective standard deviations. In so doing and by following Nijskens and Wagner (2011), we obtain a return series with a standard deviation of one. By this means, the beta term in equation (1) becomes a measure of the correlation of the original series. This normalization can be implemented in the modified regression model, Equation (A6), where the symbol ~ represents the transformed series:

$\tilde{R}_{i,t}=\rho_{i,0}+\rho_{i,1}\tilde{R}_{m,i,t}+ \rho_{i,2}\left[ \left( T_{1B}-t \right)\left( t-T_{2B} \right)D_{11,t}+\left( T_{1S}-t \right)\left( t-T_{2S} \right){F_{i}D}_{12,t} \right]\tilde{R}_{m,i,t}+\rho_{i,3}\left[ \left( t-T_{1B} \right)(D_{11,t}+D_{12,t})+\left( T_{2S}-T_{1B} \right)D_{2,t} \right]\tilde{R}_{m,i,t}+\epsilon_{i,t}$ (A8)

where:

$$\tilde{R}_{i,t}=\left\{ \begin{aligned} \frac{\text{R}_{\text{i,t}}}{\text{σ}_{\text{i,t<}\text{T}_{\text{1B}}}}\text{if t<}\text{T}_{\text{1B}} \\ \frac{\text{R}_{\text{i,t}}}{\text{σ}_{\text{i,}\text{T}_{\text{1B}}\text{≤t≤}\text{T}_{\text{2S}}}}\text{if }\text{T}_{\text{1B}}\text{ ≤t≤}\text{T}_{\text{2S}} \\ \frac{\text{R}_{\text{i,t}}}{\text{σ}_{\text{i,t>}\text{T}_{\text{2S}}}}\text{if t>}\text{T}_{\text{2S}} \end{aligned} \right.$$

$$\tilde{R}_{m,t}=\left\{ \begin{aligned} \frac{R_{m,t}}{\sigma_{m,t<T_{1B}}}if t<T_{1B} \\ \frac{R_{m,t}}{\sigma_{m,T_{1B}\leq t\leq T_{2S}}}if T_{1B}\leq t\leq T_{2S} \\ \frac{R_{m,t}}{\sigma_{m,t>T_{2S}}}if t>T_{2S} \end{aligned} \right.$$

and

$F_{i}=\frac{\frac{\rho_{i,3}^{2}}{4\rho_{i,2}}+\rho_{i,2}T_{2B}^{2}+\rho_{i,3}T_{2B}}{\frac{\rho_{i,3}^{2}}{4\rho_{i,2}}+\rho_{i,2}T_{2S}^{2}+\rho_{i,3}T_{2B}}$ (A9)

In the computation of the normalized variables, we allow standard deviations to differ before, during and after the event period.

This equation, as with equation (A6), is estimated by assuming that the variance in the error term,$\epsilon_{i,t}$, follows a GARCH process (1,1). The results of the estimation allow us to see the beta correlation component and its possible variation as a consequence of securitization.

To evaluate how much of the variation in the beta is due to the correlation effect (${\Delta\rho}_{i,m}$) and how much to the bank’s standard deviation relative to the market ($\Delta\frac{\sigma_{i}}{\sigma_{m}}$), we derive an expression for the change in the relative standard deviation. In which 0 indicates the period immediately prior to the event window and 1, the period that comes directly after. Therefore, the systematic risk after the event may be expressed as:

$\beta_{i}^{1} =\rho_{i,m}^{1}\frac{\sigma_{i}^{1}}{\sigma_{m}^{1}}=\left( \rho_{i,m}^{0}+\Delta\rho_{i,m} \right)\frac{\sigma_{i}^{1}}{\sigma_{m}^{1}}=\beta_{i}^{0}+\Delta\beta_{i}$ (A10)

Therefore:

$\frac{\sigma_{i}^{1}}{\sigma_{m}^{1}}=\frac{\beta_{i}^{0}+\Delta\beta_{i}}{\rho_{i,m}^{0}+\Delta\rho_{i,m}}$ (A11)

Reordering, we obtain the equation for the change in the relative standard deviation:

$\Delta\frac{\sigma_{i}}{\sigma_{m}}=\frac{\sigma_{i}^{1}}{\sigma_{m}^{1}}-\frac{\sigma_{i}^{0}}{\sigma_{m}^{0}}=\frac{\beta_{i}^{0}+\Delta\beta_{i}}{\rho_{i,m}^{0}+\Delta\rho_{i,m}}-\frac{\beta_{i}^{0}}{\rho_{i,m}^{0}}$ (A12)

which is obtained from the estimated coefficients of the equations (A6) and (A8)

**Table AI**

Descriptive statistics of bank stock returns.

| **Banks** | **S** | **K** | **JB** | **p-value** | **ADF** | **p-value** | **Q(r_t_^2^)** | **p-value** | **Q(\|r_t_\|)** | **p-value** | **N. obs** |
| --- | --- | --- | --- | --- | --- | --- | --- | --- | --- | --- | --- |
| ***Spanish Banks***  *Banco de Andalucía* | -0.1 | 18.8 | 27865.6 | 0.0 | -56.1 | 0.0 | 93.4 | 0.0 | 156.5 | 0.0 | 2659 |
| *Banesto* | 0.5 | 17.3 | 31247.2 | 0.0 | -59.4 | 0.0 | 77.5 | 0.0 | 264.5 | 0.0 | 3634 |
| *Bankinter* | 0.7 | 7.7 | 4180.7 | 0.0 | -60.1 | 0.0 | 170.4 | 0.0 | 218.2 | 0.0 | 4043 |
| *BBVA* | 0.6 | 9.5 | 7268.8 | 0.0 | -60.1 | 0.0 | 81.8 | 0.0 | 204.3 | 0.0 | 4043 |
| *Banco de Castilla* | 0.2 | 16.6 | 19262.1 | 0.0 | -57.5 | 0.0 | 36.7 | 0.0 | 87.9 | 0.0 | 2494 |
| *Banco de Crédito Balear* | 1.9 | 50.5 | 236284.1 | 0.0 | -55.2 | 0.0 | 12.9 | 0.0 | 66.2 | 0.0 | 2494 |
| *Banco de Galicia* | 0.0 | 10.0 | 5134.6 | 0.0 | -36.1 | 0.0 | 57.1 | 0.0 | 96.2 | 0.0 | 2494 |
| *Banco Guipuzcoano* | 0.8 | 12.8 | 12455.5 | 0.0 | -58.2 | 0.0 | 125.1 | 0.0 | 253.6 | 0.0 | 2986 |
| *Banco Pastor* | 1.1 | 17.2 | 28878.3 | 0.0 | -55.3 | 0.0 | 46.5 | 0.0 | 209.4 | 0.0 | 3334 |
| *Banco Popular Español* | 0.6 | 9.8 | 8249.3 | 0.0 | -60.3 | 0.0 | 60.1 | 0.0 | 219.4 | 0.0 | 4043 |
| *Banco Sabadell* | 0.9 | 13.1 | 15607 | 0.0 | -53.6 | 0.0 | 74.7 | 0.0 | 160.8 | 0.0 | 3551 |
| *Banco Santander* | 0.2 | 11.1 | 13677.8 | 0.0 | -71.1 | 0.0 | 81.8 | 0.0 | 290 | 0.0 | 4982 |
| *Banco de Valencia* | 8.1 | 463.9 | 32703732 | 0.0 | -44.3 | 0.0 | 1.9 | 0.1 | 309.4 | 0.0 | 3689 |
| *Banco de Vasconia* | -0.1 | 20.7 | 32674.4 | 0.0 | -59.0 | 0.0 | 133.3 | 0.0 | 130.7 | 0.0 | 2494 |
| *Bankia* | -0.6 | 26.01 | 19372.18 | 0.0 | -20.9 | 0.0 | 34.5 | 0.0 | 165.2 | 0.0 | 875 |
| *Caixa Bank* | 0.4 | 8.6 | 2523.1 | 0.0 | -43.9 | 0.0 | 27.2 | 0.0 | 46.4 | 0.0 | 1861 |
| ***British Banks***  *3i Group* | 0.2 | 11.1 | 10759.6 | 0.0 | -60.9 | 0.0 | 91.2 | 0.0 | 264.2 | 0.0 | 3911 |
| *Barclays* | 3.5 | 89.9 | 1238985. | 0.0 | -60.9 | 0.0 | 10.1 | 0.0 | 324.0 | 0.0 | 3911 |
| *BlackRock Throgmorton Trust* | -0.5 | 10.8 | 10088.43 | 0.0 | -39.3 | 0.0 | 333.5 | 0.0 | 359.7 | 0.0 | 3911 |
| *Henderson Group* | 0.7 | 13.9 | 14779.3 | 0.0 | -54.3 | 0.0 | 105.3 | 0.0 | 156.4 | 0.0 | 2876 |
| *HSBC Holdings* | 0.04 | 15.5 | 25813.3 | 0.0 | -47.5 | 0.0 | 125.7 | 0.0 | 315.0 | 0.0 | 3911 |
| *Intermediate Capital Group* | 0.5 | 23.7 | 70279.6 | 0.0 | -57.9 | 0.0 | 14.9 | 0.0 | 229.5 | 0.0 | 3911 |
| *Investec* | 0.4 | 11.9 | 10906.2 | 0.0 | -13.9 | 0.0 | 87.7 | 0.0 | 220.4 | 0.0 | 3247 |
| *Lloyds Banking Group* | 0.7 | 45.3 | 371687.0 | 0.0 | -30.5 | 0.0 | 188.9 | 0.0 | 670.8 | 0.0 | 4981 |
| *London Capital Group Holdings* | 0.2 | 19.6 | 27239.1 | 0.0 | -42.3 | 0.0 | 72.3 | 0.0 | 146.3 | 0.0 | 2354 |
| *Paragon Group* | 0.0 | 53.8 | 536020.7 | 0.0 | -42.1 | 0.0 | 613.7 | 0.0 | 603.3 | 0.0 | 4981 |
| *Royal Bank of Scotland* | -1.8 | 65.1 | 630618.4 | 0.0 | -44.3 | 0.0 | 37.7 | 0.0 | 424.7 | 0.0 | 3911 |
| *Standard Chartered* | 0.7 | 16.9 | 32172.2 | 0.0 | -47.9 | 0.0 | 165.9 | 0.0 | 420.4 | 0.0 | 3911 |
| *Abbey plc* | 1.8 | 36.7 | 188205.6 | 0.0 | -55.7 | 0.0 | 28.9 | 0.0 | 104.4 | 0.0 | 3911 |
| *Virgin Money Holding* | -2.2 | 30.7 | 31103.5 | 0.0 | -18.3 | 0.0 | 239.1 | 0.0 | 120.8 | 0.0 | 949 |
| ***Austrian Banks***  *Erste group* | 0.08 | 10.2 | 8656.4 | 0.0 | -45.2 | 0.0 | 303.8 | 0.0 | 344.0 | 0.0 | 3912 |
| *Immofinanz AG* | 0.3 | 80.3 | 974053.7 | 0.0 | -8.9 | 0.0 | 1134.8 | 0.0 | 1448 | 0.0 | 3912 |
| ***French Banks***  *BNPParibas* | 0.6 | 12.4 | 14739.9 | 0.0 | -38.9 | 0.0 | 60.9 | 0.0 | 258.7 | 0.0 | 3912 |
| *Natixis* | 1.4 | 25.2 | 81693.5 | 0.0 | -60.5 | 0.0 | 58.5 | 0.0 | 388.3 | 0.0 | 3912 |
| *Societe generale* | 0.4 | 10.1 | 8299.5 | 0.0 | -58.7 | 0.0 | 102.3 | 0.0 | 266.1 | 0.0 | 3912 |

Table AI (continued). Descriptive statistics of bank stock returns

| **Banks** | **S** | **K** | **JB** | **p-value** | **ADF** | **p-value** | **Q(r_t_^2^)** | **p-value** | **Q(\|r_t_\|)** | **p-value** | **N. obs** |
| --- | --- | --- | --- | --- | --- | --- | --- | --- | --- | --- | --- |
| ***Italian Banks***  *Banca Nazionale del Lavoro* | 0.6 | 8.8 | 2977.2 | 0.0 | -43.9 | 0.0 | 35.1 | 0.0 | 123.2 | 0.0 | 1972 |
| *Banca Italease* | 1.6 | 29.4 | 37005.5 | 0.0 | -31.5 | 0.0 | 84.7 | 0.0 | 150.4 | 0.0 | 1253 |
| *Banca Monte dei Paschi di Siena* | 0.1 | 10.7 | 9739.5 | 0.0 | -57.6 | 0.0 | 41.8 | 0.0 | 222.1 | 0.0 | 3875 |
| *Banca popolare dell Emilia Romagne* | 0.4 | 10.6 | 9463.5 | 0.0 | -74.5 | 0.0 | 197.2 | 0.0 | 587.4 | 0.0 | 3876 |
| *Crédito Emiliano* | 0.4 | 8.0 | 4419.5 | 0.0 | -75.3 | 0.0 | 106.9 | 0.0 | 186.6 | 0.0 | 4046 |
| *Banca Carige* | - 0.1 | 13.1 | 17077.2 | 0.0 | -82.6 | 0.0 | 102.0 | 0.0 | 477.5 | 0.0 | 4045 |
| *Banca Ifis* | -0.8 | 21.1 | 47199.6 | 0.0 | -83.7 | 0.0 | 6.6 | 0.0 | 97.3 | 0.0 | 3412 |
| *Intesa Sanpaolo* | 0.16 | 8.8 | 5794.7 | 0.0 | -32 | 0.0 | 115.3 | 0.0 | 217.1 | 0.0 | 4047 |
| *Meliorbanca* | 0.11 | 8.1 | 2319.1 | 0.0 | -46.8 | 0.0 | 69.8 | 0.0 | 121.4 | 0.0 | 2144 |
| *Banca Popolare dell Etruria e del Lazio* | 0.4 | 8.9 | 5492.3 | 0.0 | -59.9 | 0.0 | 142.4 | 0.0 | 251.5 | 0.0 | 3625 |
| *Banca Popolare di Intra* | 0.01 | 16.8 | 19922.8 | 0.0 | -51.6 | 0.0 | 19.2 | 0.0 | 161.5 | 0.0 | 2489 |
| *Banca Popolare di Milano* | -3.5 | 98.9 | 1561064. | 0.0 | -60.3 | 0.0 | 5.2 | 0.02 | 236.4 | 0.0 | 4047 |
| *Banca popolare di Spoleto* | 1.3 | 18.7 | 39845.2 | 0.0 | -65.3 | 0.0 | 367.1 | 0.0 | 249.1 | 0.0 | 3733 |
| *Unicredit* | 0.2 | 10.6 | 9964.6 | 0.0 | -31.6 | 0.0 | 245.6 | 0.0 | 425.6 | 0.0 | 4047 |
| ***Portuguese Banks***  *Banco Comercial Portugues* | 0.7 | 14.7 | 23777.2 | 0.0 | -58.2 | 0.0 | 70.9 | 0.0 | 380.3 | 0.0 | 4047 |
| *Banco Espiritu Santo* | -2.4 | 58.4 | 510837.4 | 0.0 | -49.9 | 0.0 | 974.9 | 0.0 | 800.1 | 0.0 | 3959 |
| *Banco Santander Totta* | 0.4 | 9.8 | 4335.6 | 0.0 | -44.6 | 0.0 | 30.4 | 0.0 | 75.1 | 0.0 | 2201 |
| *Banco Portugues de Investimento* | 0.9 | 13.4 | 19196.1 | 0.0 | -59.9 | 0.0 | 120.2 | 0.0 | 411.5 | 0.0 | 4047 |
| *Banco Intern de Funchal* | -0.1 | 47.0 | 62980.7 | 0.0 | -28.6 | 0.0 | 65.2 | 0.0 | 142.6 | 0.0 | 779 |
| ***Irish Banks***  *Permanent TSB* | 0.3 | 27.6 | 126379.3 | 0.0 | -35.30 | 0.0 | 169.7 | 0.0 | 738.7 | 0.0 | 4982 |
| ***German Banks***  *Commerzbank AG* | 0.3 | 10.4 | 9060.1 | 0.0 | -57.5 | 0.0 | 219.3 | 0.0 | 423.6 | 0.0 | 3912 |
| *Deutshe bank* | 0.5 | 12.1 | 13583.3 | 0.0 | -45.6 | 0.0 | 171.5 | 0.0 | 323.4 | 0.0 | 3912 |
| *Deutshe postbank.DE* | 0.0 | 11.0 | 9913.8 | 0.0 | -59.6 | 0.0 | 165.7 | 0.0 | 183.6 | 0.0 | 3682 |
| *Grenkeleasing* | 2.1 | 45.5 | 293490.7 | 0.0 | -62.8 | 0.0 | 349.1 | 0.0 | 379.9 | 0.0 | 3846 |
| *HSBC trinkaus* | 0.8 | 19.8 | 46632.9 | 0.0 | -32.5 | 0.0 | 56.4 | 0.0 | 248.6 | 0.0 | 3910 |
| ***Dutch Banks***  *Delta LLoyd* | 0.29 | 19.5 | 22427.1 | 0.0 | -27.8 | 0.0 | 14.3 | 0.0 | 132.7 | 0.0 | 1976 |
| *ING group NV* | 0.6 | 18.5 | 49924.9 | 0.0 | -44.3 | 0.0 | 463.7 | 0.0 | 741.4 | 0.0 | 4982 |
| ***Danish Banks***  *Danske bank* | 0.17 | 8.8 | 5607.6 | 0.0 | -59.4 | 0.0 | 376.0 | 0.0 | 435.1 | 0.0 | 3912 |

Note: S is the skewness and K is the kurtosis; JB denotes the Jarque–Bera normality test; ADF is the augmented Dickey–Fuller unit root test with lags for the auxiliary equation determined using the Schwarz criterion; Q(r_t_^2^) and Q(|r_t_|) are the Ljung–Box tests for the first-order autocorrelation of the squares and the absolute values of the return respectively. The data used correspond to the period 6/01/1999-4/07/2018.
